# Socioeconomic position and use of healthcare in the last year of life: A systematic review and meta-analysis

**DOI:** 10.1371/journal.pmed.1002782

**Published:** 2019-04-23

**Authors:** Joanna M. Davies, Katherine E. Sleeman, Javiera Leniz, Rebecca Wilson, Irene J. Higginson, Julia Verne, Matthew Maddocks, Fliss E. M. Murtagh

**Affiliations:** 1 Cicely Saunders Institute of Palliative Care, Policy and Rehabilitation, King’s College London, London, United Kingdom; 2 Health Intelligence, Public Health England, Bristol, United Kingdom; 3 Wolfson Palliative Care Research Centre, Hull York Medical School, University of Hull, Hull, United Kingdom; Weill Cornell Medical College, UNITED STATES

## Abstract

**Background:**

Low socioeconomic position (SEP) is recognized as a risk factor for worse health outcomes. How socioeconomic factors influence end-of-life care, and the magnitude of their effect, is not understood. This review aimed to synthesise and quantify the associations between measures of SEP and use of healthcare in the last year of life.

**Methods and findings:**

MEDLINE, EMBASE, PsycINFO, CINAHL, and ASSIA databases were searched without language restrictions from inception to 1 February 2019. We included empirical observational studies from high-income countries reporting an association between SEP (e.g., income, education, occupation, private medical insurance status, housing tenure, housing quality, or area-based deprivation) and place of death, plus use of acute care, specialist and nonspecialist end-of-life care, advance care planning, and quality of care in the last year of life. Methodological quality was evaluated using the Newcastle-Ottawa Quality Assessment Scale (NOS). The overall strength and direction of associations was summarised, and where sufficient comparable data were available, adjusted odds ratios (ORs) were pooled and dose-response meta-regression performed.

A total of 209 studies were included (mean NOS quality score of 4.8); 112 high- to medium-quality observational studies were used in the meta-synthesis and meta-analysis (53.5% from North America, 31.0% from Europe, 8.5% from Australia, and 7.0% from Asia). Compared to people living in the least deprived neighbourhoods, people living in the most deprived neighbourhoods were more likely to die in hospital versus home (OR 1.30, 95% CI 1.23–1.38, *p* < 0.001), to receive acute hospital-based care in the last 3 months of life (OR 1.16, 95% CI 1.08–1.25, *p* < 0.001), and to not receive specialist palliative care (OR 1.13, 95% CI 1.07–1.19, *p* < 0.001). For every quintile increase in area deprivation, hospital versus home death was more likely (OR 1.07, 95% CI 1.05–1.08, *p* < 0.001), and not receiving specialist palliative care was more likely (OR 1.03, 95% CI 1.02–1.05, *p* < 0.001). Compared to the most educated (qualifications or years of education completed), the least educated people were more likely to not receive specialist palliative care (OR 1.26, 95% CI 1.07–1.49, *p* = 0.005).

The observational nature of the studies included and the focus on high-income countries limit the conclusions of this review.

**Conclusions:**

In high-income countries, low SEP is a risk factor for hospital death as well as other indicators of potentially poor-quality end-of-life care, with evidence of a dose response indicating that inequality persists across the social stratum. These findings should stimulate widespread efforts to reduce socioeconomic inequality towards the end of life.

## Abstract 2

Joanna Davies and colleagues highlight the association between low socioeconomic status and poor end of life care as well as increased risk of hospital death.

## Author summary

**Why was this study done?:**

- Social inequality in health is a global phenomenon; people with lower socioeconomic position (SEP) experience earlier onset of disease and have reduced life expectancy.
- Studies have identified low SEP as a risk factor for worse care at the end of life, and several socioeconomic factors have been identified as determinants of care towards the end of life.
- Despite growing recognition, no empirical synthesis of evidence exists to support efforts to reduce socioeconomic inequality at the end of life.

**What did the researchers do and find?:**

- We carried out a systematic review of studies that reported an association between a measure of SEP (including income, education, occupation, private medical insurance status, housing tenure, housing quality, or area-based deprivation) and healthcare received by adults in their last year of life (including place of death, use of acute care, use of specialist palliative care, use of nonspecialist end-of-life care, use of advance care planning, or quality of care) in high-income countries.
- A total of 209 studies were included in the review; we found consistent evidence that low SEP increases the odds of hospital versus home death and of using acute care services in the last 3 months of life and reduces the odds of using specialist palliative care in the last year of life.
- We also found that measurement of SEP in this field is dominated by measures of area deprivation and education, and justification for choice of SEP measure(s) is often inadequately described.

**What do these findings mean?:**

- We have found consistent evidence of socioeconomic inequality in the care received by people towards the end of life, in that people with lower SEP are more likely to experience worse care.
- We must now make further efforts to reduce this inequality.
- We recommend the following: that all research on care received towards the end of life should attempt to account for SEP, end-of-life care interventions should be analysed for their different effects across the social strata, and the planning and provision of end-of-life care services should consider SEP in local populations.

## Introduction

Social inequality in health status, access to, and quality of healthcare is a global phenomenon [1]. For example, in the United Kingdom, people living in the most deprived neighbourhoods (measured using the Index of Multiple Deprivation at Lower Layer Super Output Area Level) have a life expectancy up to 7 years shorter, and experience the onset of disease and disability as much as 17 years earlier, than people living in the least deprived neighbourhoods [2]. Explanations for the social determinants of health emphasise the cumulative effect of events throughout the life course on health outcomes later in life [3]. Structural (policy and culture), individual (material, behavioural, and psychosocial), and health-system factors all contribute to health inequality [3].

In high-income countries, proposed population-level quality indicators for end-of-life care include receipt of specialist palliative care, hospital admissions in the last months of life, emergency department attendance in the last months of life, and whether people are supported to be cared for in their usual place of residence rather than in hospital [4]. In Canada, the United States, and the UK, lower socioeconomic position (SEP; measured through neighbourhood deprivation) is associated with increased risk of death in hospital rather than in the community [5–7] and more emergency admissions in the last months of life [8,9]. In the UK, improvements in where people die—with fewer people dying in hospital and more at home or in hospice—have been significantly greater for those who are least deprived [10,11]. In terms of hospice deaths, the gap between the least and most deprived grew by 25% between 1993–1997 and 2008–2012 [11].

At the end of life, even within systems of universal coverage, people with limited resources generally have more complex clinical needs [12] and are less able to support their own care at home [13]. More socioeconomically deprived people may also have poor access to and knowledge of services and/or communicate their care preferences less [13–15]. One consequence of rapid population ageing is the rising numbers of deaths; globally, deaths will increase from 57 million in 2015 to 70 million over the next 15 years [16]. Social inequality in health, including at the end of life, is likely to be exacerbated by the ageing population.

While measures of SEP are commonly included as covariates in studies about end-of-life care, there is considerable variation in what is measured and how. Information on social inequality at the end of life has not been systematically summarised, including on the magnitude of effect of SEP on outcomes. This limits our understanding of how SEP relates to end-of-life care, as well as the incorporation of SEP into quality evaluations and service delivery plans.

The aim of this review is to systematically identify, synthesise, and quantify existing evidence on the association between SEP and use of healthcare in the last year of life—including place of death, use of acute care use, use of specialist palliative care and nonspecialist end-of-life care, use of advance care planning, and quality of care—and to report how SEP has been measured within this literature.

## Methods

The protocol was registered (CRD42017055686) with PROSPERO, the international prospective register of systematic reviews [17], and the study was conducted and reported following the Preferred Reporting Items for Systematic Reviews and Meta-Analyses (PRISMA) statement (S1 PRISMA Checklist) [18] and MOOSE [19] guidelines for meta-analysis and systematic reviews of observational studies. Ethical approval was not required for this review.

### Search strategy

The following databases were searched from inception to 1 February 2019: MEDLINE, MEDLINE in process, EMBASE, PsycINFO, CINAHL, and ASSIA. Search terms including subject headings and free-text words were developed in MEDLINE and then adapted for other databases (S1 Text). Key papers identified from reviews by Henson (2015) [20–24] and Gomes (2006) [7,25–29], as well as prior knowledge [30–34], were used to refine the search terms. Consultation with the review team (FM, MM, KS, and IH) provided expert advice to identify missing papers and relevant reviews [35–47], the reference lists of which were searched manually. No language restrictions were applied, non–English-language papers were assessed for inclusion, and data were extracted by a native speaker. Grey literature and thesis and conference abstracts were included, and requests for additional data were made to authors by email.

### Study inclusion criteria

We included empirical observational studies reporting an association between SEP and healthcare received by adults (≥18 years old) in the last year of life. Studies were restricted to those from high-income countries to limit contextual differences in the availability of services and strengthen assumptions made about preferable service-use outcomes [48]. Studies were included if they met the following criteria.

- Participants were adults with malignant and nonmalignant advanced or incurable illness in community or inpatient settings receiving or not receiving specialist palliative care, and at least 80% of the sample were in the last year of life (based on date of death or clinical prognosis).
- An indicator of SEP was reported, such as income, education, occupation, private medical insurance status, housing tenure, housing quality, or area-based deprivation. Race and ethnicity are conceptually separate constructs than that of SEP and beyond the scope of this review [49,50].
- At least one of the following comprehensive set of outcomes was reported: place of death, acute care admission, use of specialist palliative care, use of nonspecialist end-of-life care, use of advance care planning, or quality of care. Outcomes were selected based on prior knowledge of availability within the literature. Patient-reported or patient-centred outcome measures were included as indicators of quality of care [51,52].
- The study design was empirical and observational, either prospective or retrospective; experimental, qualitative, or case-study designs were not suited to the review aims and were excluded. Area-level studies, in which the unit of analysis was not individuals, were also excluded.

### Study selection

Study selection and de-duplication was managed in EndNote X8 (Clarivate Analytics, Philadelphia, PA) (JD). Titles and abstracts retrieved from the electronic database and reference list searches were first screened, and then full texts of potentially eligible studies were sourced and reviewed independently by 2 authors (JD plus MM, JL, or RW). Disagreement was resolved through discussion with a third author (KS). Multiple studies based on the same sample of individuals were treated as duplicates; inclusion was prioritised based on larger sample size and study quality.

### Quality evaluation and grading of evidence

**Fig 1:**
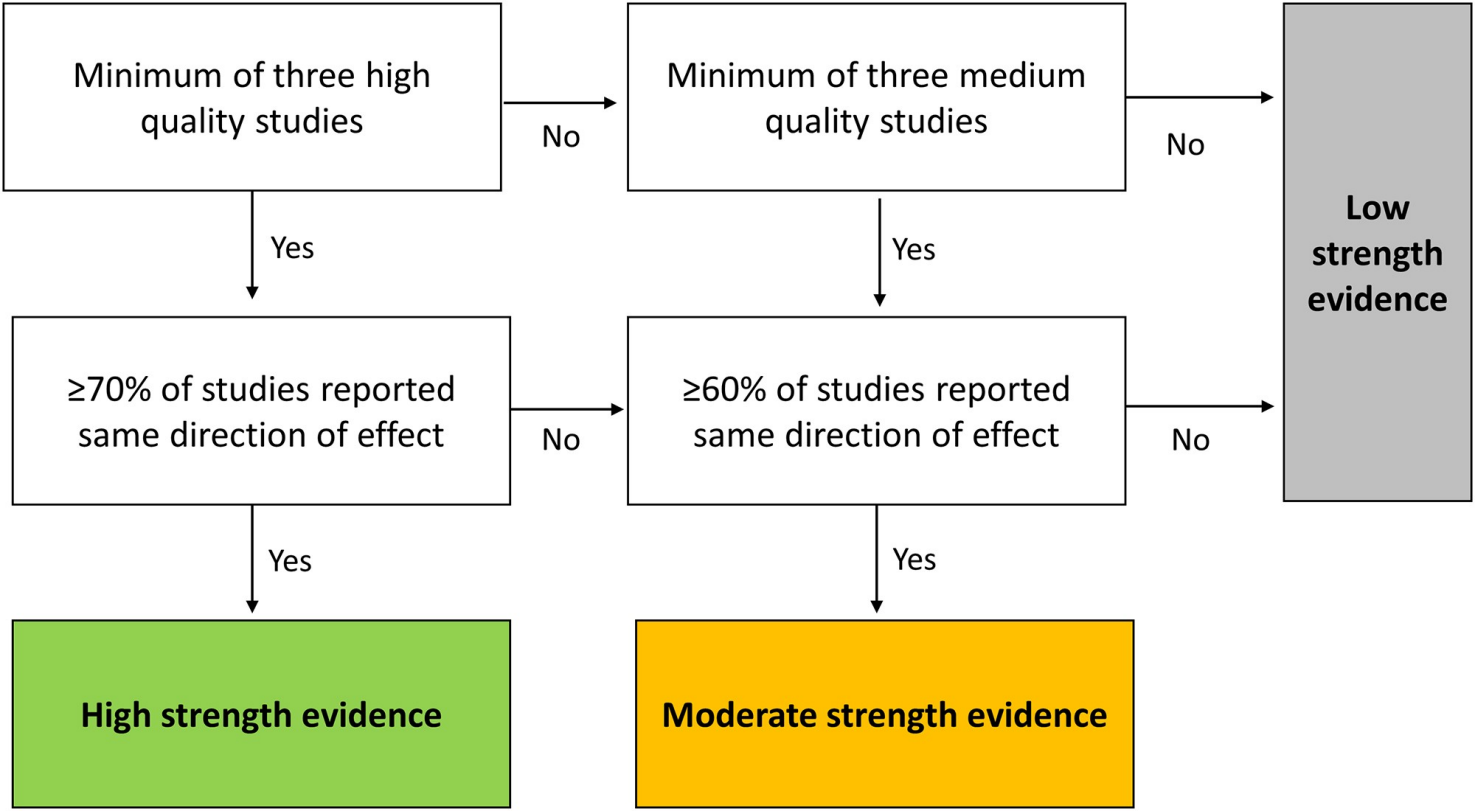
Algorithm for evaluating overall strength of evidence for each combination of SEP exposure and outcome, based on quality, quantity, and consistency of the evidence [54]. High-quality studies were those that had controlled for age and sex in a multivariable analysis and had an NOS score of ≥7. Medium quality was assigned to studies that had controlled for age and/or sex in a multivariable analysis and had an NOS score of ≥5, or had not carried out multivariable analysis but had an NOS score of ≥6. Low-quality studies had no multivariable analysis and an NOS score of ≤5, or an NOS score of ≤4. High-strength evidence required ≥70% agreement about the direction of the exposure outcome association; moderate strength evidence required ≥60% agreement. NOS, Newcastle-Ottawa Quality Assessment Scale; SEP, socioeconomic position.

Study quality was evaluated using the Newcastle-Ottawa Quality Assessment Scale (NOS) [53], a 9-item measure developed for observational studies with a focus on sample representativeness, data quality, and appropriateness of analysis. Aligned with the aims of this review, representativeness of the exposed cohort (NOS item 1 ‘selection’) was judged to be of high quality if the sample reflected the socioeconomic strata in the country of origin. On this basis, national population-based or nationally representative samples not restricted by demographics, diagnosis, or geography were considered to have a low sampling bias and be of the highest quality. Studies that adjusted for confounders—thus limiting bias—were also of higher quality, with age and sex being the factors considered most important to control for (NOS item 5 ‘comparability’). Appraisal of articles was carried out independently by two authors (JD and MM), with disagreement resolved through discussion. The overall strength of the evidence and the direction of the association between SEP exposures and outcomes were graded using an adaptation of a previously established algorithm (Fig 1) [54], taking into account 3 key elements important for grading studies—quality, quantity, and consistency [55].

### Data extraction

A piloted data extraction form was used to extract relevant information from included studies. Data items included study characteristics, sample characteristics, type of SEP measure used, and adjusted estimates. Data were extracted by one author (JD), and then two authors (JL and RW) independently checked a 20% sample for accuracy; errors were verified through discussion and were corrected.

Most studies reported adjusted odds ratios (ORs). For studies reporting risk ratios (RRs), when possible, an OR was derived as a function of the RR, proportion of cases, and proportion of exposed [56]. Most studies treated SEP exposures as categorical. Some studies treated SEP exposures as numerical or ordinal, reporting a single effect size for a unit change in the exposure. Those based on a numeric scale, e.g., income in US dollars, could not be converted to ORs. Those based on underlying categories were converted by raising the effect size to the number of categories separating the highest and lowest. For example, for a study reporting a unit-change OR of 0.92 going from high to low SEP with 5 categories, the OR for the lowest SEP group was approximated by raising 0.92 to the power of 4 (0.92 × 0.92 × 0.92 × 0.92 = 0.72).

### Synthesis and statistical analysis

Studies were initially grouped according to outcome and exposure categories. To avoid double counting samples, each study contributed no more than once in each category. Within-study duplicates—e.g., if two area-based measures were used—were prioritised based on heterogeneity with other measures. Most outcomes were defined in terms of receipt or not of a service. For place of death, death at home, in hospice, or in long-term care (LTC) was considered favourable compared to death in hospital, in line with evidence on preferences for place of death in high-income countries [57]. SEP measures were described in terms of the average number used across studies; how the measures were constructed as binary, categorical, or continuous variables; and whether they were objective or subjective measures [50].

The meta-analysis was restricted to high- and medium-quality studies (see Fig 1 for definition) that had used multivariable analysis to reduce bias from confounding, and were more likely to be representative of the population social strata from which the study samples were drawn. Following an approach used elsewhere [58,59], the adjusted OR for the lowest versus the highest SEP group was presented. ORs were standardised so that an OR > 1 indicated a pro–high-SEP association. The overall strength and direction of the evidence was summarised using a diagram influenced by an existing design [60,61]. Rules for deriving the strength of evidence are described in the algorithm in Fig 1. Direction was determined by categorising associations as either ‘pro–high-SEP’ or ‘pro–low-SEP’. The interpretation of null effects relies more heavily on sample size, which is not incorporated in the diagram; therefore, following peer review that highlighted this limitation, null effects were not depicted.

For subgroups of exposure and outcome, when enough comparable studies were available presenting OR for categories of SEP exposure, adjusted ORs were pooled using random-effects models. Acute care studies were only pooled if they were about care received in last 3 months of life to reflect established aggressive care definitions [62]. For better comparability, studies about use of nonspecialist end-of-life care were pooled only when the outcome was the receipt of nursing or support worker homecare. High levels of heterogeneity were expected given the observational nature of the studies and variety in measurement of SEP and definition of outcomes; heterogeneity was reported using Higgins’ I-squared (*I*^2^).[63] Studies reporting separate estimates for subgroups of the same sample, by year [64], gender [65], diagnosis [66], or regions within a country [67,68], were pooled prior to meta-analysis with fixed or random effects depending on level of heterogeneity (*I*^2^). Following peer review that highlighted an inconsistency with the approach taken to other subgroups, studies reporting estimates separately for people living in the community and inpatient locations were also pooled prior to meta-analysis [31,69–72]. We anticipated between-country variations due to cultural differences in end-of-life practices, particularly in Asian countries [73]. For each pooled meta-analysis, a sensitivity analysis was carried out using subgroups by country to explore differences. Further sensitivity analysis examined change in the pooled estimates after each of the studies was removed from the meta-analysis.

To examine for a dose response for studies that presented data on at least 3 exposure categories, we used a random-effects weighted meta-regression of the log-OR to derive an estimation of the summarised dose response using the glst command in Stata (Stata SE version 13; StataCorp, College Station, TX) [74–76]. This approach uses a two-stage generalised least squares model that first estimates the within-study trend and then pools these to give an overall trend estimate [76]. Using the method described by Hamling and colleagues [77], studies were first standardised so that the reference group was always the least deprived. Dose was assigned using the cumulative mean relative rank for each SEP group reported. For example, for area deprivation, if quintile 5 (the least deprived group) accounted for 33% of the sample and quintile 4 accounted for a further 25% of the sample, the mean relative rank for quintile 5 would be 17 and for quintile 4 would be 29 [58]. Dose was then centred for each study so that the dose for the reference group was 0. Doses for all groups (other than the reference group) and the corresponding log-odds were plotted and inspected visually for linearity. For studies reporting multiple estimates for subgroups of the same sample, only the largest subgroup was included because of inability to control for dependence.

## Results

**Fig 2:**
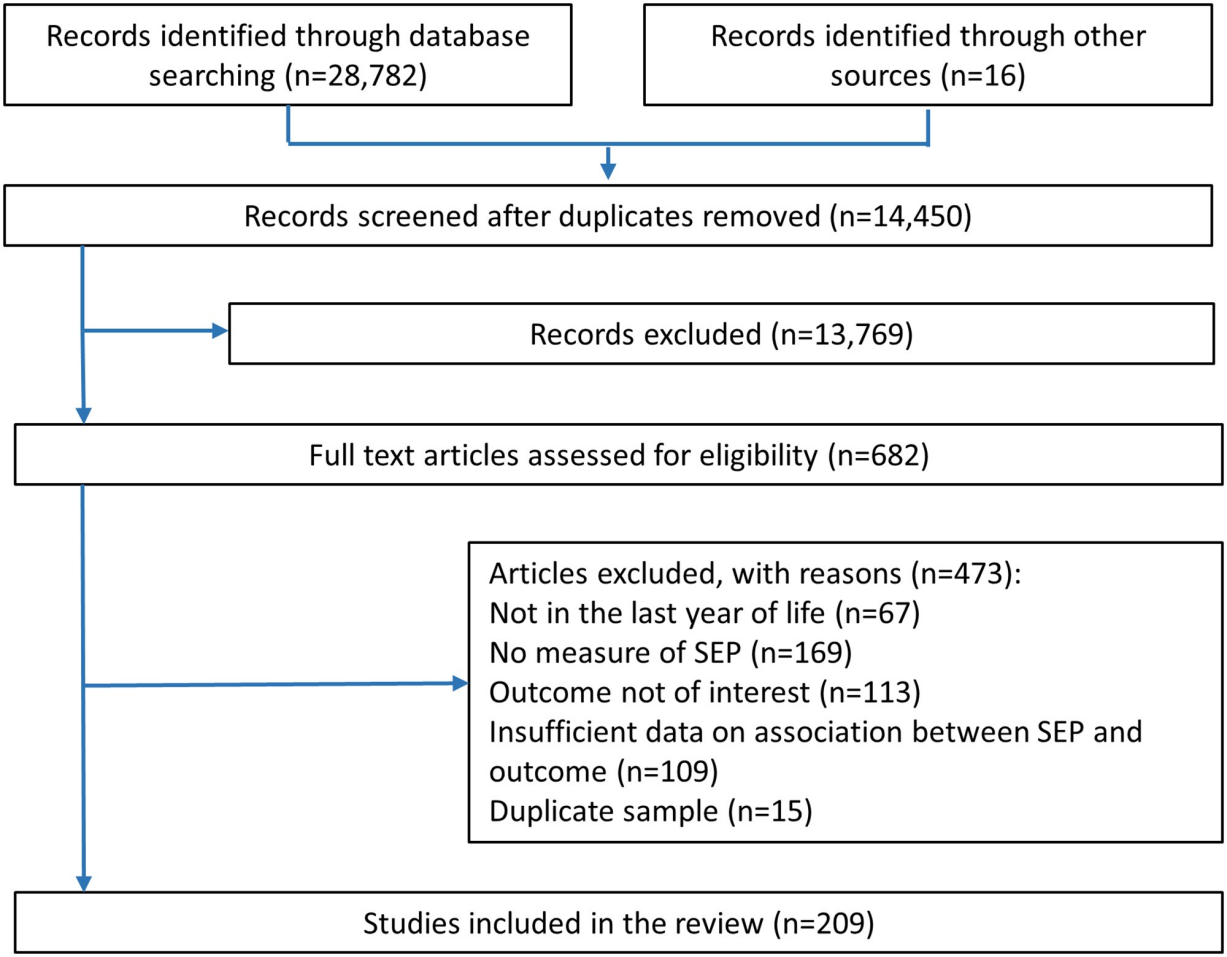
PRISMA flow diagram of papers reporting numbers of included and excluded texts. **PRISMA**, Preferred Reporting Items for Systematic Reviews and Meta-Analyses; SEP, socioeconomic position.

A total of 682 full-text articles were screened for eligibility, of which 209 were included in the review (Fig 2).

### Summary of included studies

Of the 209 studies included, 158 (75.6%) used 1 type of SEP measure; the mean number of measures per study was 1.3, and the maximum number was 6 [78]. A total of 273 SEP measurements were reported across 209 studies; these were categorised as area deprivation (29.7%), education (28.9%), income (16.8%), insurance (12.8%), occupation (4.4%), housing (3.3%), social class (3.7%), and literacy (0.4%). Of the 209 studies, 205 (98.1%) used objective measures of SEP, such as self-reported level of education or income, information obtained from administrative records, or area-based deprivation. Four studies [79–82] employed subjective measures, all concerning self-rated financial security; 172 (82.3%) studies provided a full or partial description of the SEP measure(s), including referencing the source of the measure, referencing the wording of the question used, or identifying the data set the SEP measure was contained within. In 27 (12.9%) studies, SEP was the main exposure variable of interest; 12 of these provided theoretical justification for choice of SEP measure.

The mean NOS quality score across all studies was 4.8 (range: 0–10) (see S1 Fig for histogram of scores); 97 (46.4%) of 209 studies were rated low quality, including all 11 studies on quality of care. A complete list of the low-quality studies is provided in S2 Text. The remaining 112 high- and medium-quality studies were included in further synthesis and meta-analysis (Table 1 and S3 Text). Combined, these studies report 142 outcomes of interest: most commonly, place of death (50.7%), then 25.4% on use of specialist palliative, 13.4% on use of acute care services, 7.7% on use of nonspecialist end-of-life care, and 2.8% on use of advance care planning. The majority of data were from the US (34.5%), Canada (19.0%), or Europe (not including the UK) (21.1%); 9.9% were from the UK, 8.5% from Australia, 4.2% from South Korea, 2.1% from Taiwan, and 0.7% from Singapore. Eight full-text non–English-language papers were assessed for eligibility: 2 in Japanese, 1 in German, 1 in Korean, and 3 in Spanish—3 of these were included in the review [83–85]. Data extraction was accurate for 98.1% of items.

**Table 1:** Summary of 112 high- and medium-quality studies.

	Number of studies reporting each outcome					
	Place of death (n = 72)^☯^	Acute care (*n* = 19)	Specialist palliative care (*n* = 36)	Nonspecialist end-of-life care (*n* = 11)	Advance care planning (*n* = 4)	All (*n* = 142 outcomes, reported in 112 studies)
Type of SEP measure,* ***n* (%)**						
**Income**	7 (8.6)	2 (10.5)	5 (10.9)	1 (8.3)	2 (25.0)	17 (10.2)
**Education**	27 (33.3)	1 (5.3)	5 (10.9)	2 (16.7)	4 (50.0)	39 (23.5)
**Private insurance status**	6 (7.4)	2 (10.5)	10 (21.7)	4 (33.3)	1 (12.5)	23 (13.9)
**Housing**	4 (4.9)	0 (0)	0 (0)	0 (0)	0 (0)	4 (2.4)
**Area deprivation**	33 (40.7)	14 (73.7)	26 (56.5)	5 (41.7)	1 (12.5)	79 (47.6)
**Occupation**	4 (4.9)	0 (0)	0 (0)	0 (0)	0 (0)	4 (2.4)
**Country/region, *n* (%)**						
**UK**	11 (15.3)	3 (15.8)	0 (0)	0 (0)	0 (0)	14 (9.9)
**Europe**	22 (30.6)	1 (5.3)	3 (8.3)	4 (36.4)	0 (0)	30 (21.1)
**US**	15 (20.8)	5 (26.3)	23 (63.9)	3 (27.3)	3 (75.0)	49 (34.5)
**Canada**	10 (13.9)	7 (36.8)	6 (16.7)	4 (36.4)	0 (0)	27 (19.0)
**Australia**	6 (8.3)	2 (10.5)	4 (11.1)	0 (0)	0 (0)	12 (8.5)
**Asia**	8 (11.1)	1 (5.3)	0 (0)	0 (0)	1 (25.0)	10 (7.0)
**Outcomes on cancer**						
**patients only, *n* (%)**	34 (47.2)	9 (47.4)	22 (61.1)	6 (54.5)	0 (0)	63 (44.4)
**Study time period**						
**(range of years)**	1979–2015	1992–2015	1990–2014	1991–2013	1986–2013	1979–2015
**Study design, *n* (%)**						
**Prospective**	2 (2.8)	0 (0)	1 (2.8)	1 (9.1)	0 (0)	4 (2.8)
**Retrospective**	70 (97.2)	19 (100)	35 (97.2)	10 (90.9)	4 (100)	138 (97.2)

^☯^From 64 studies (outcomes on multiple countries presented in the same study are counted separately).

*Within-column totals sum to more than the number of outcomes because each study can report data on multiple SEP exposures.

**Abbreviation:** SEP, socioeconomic position.

### Strength and direction of the evidence

**Fig 3:**
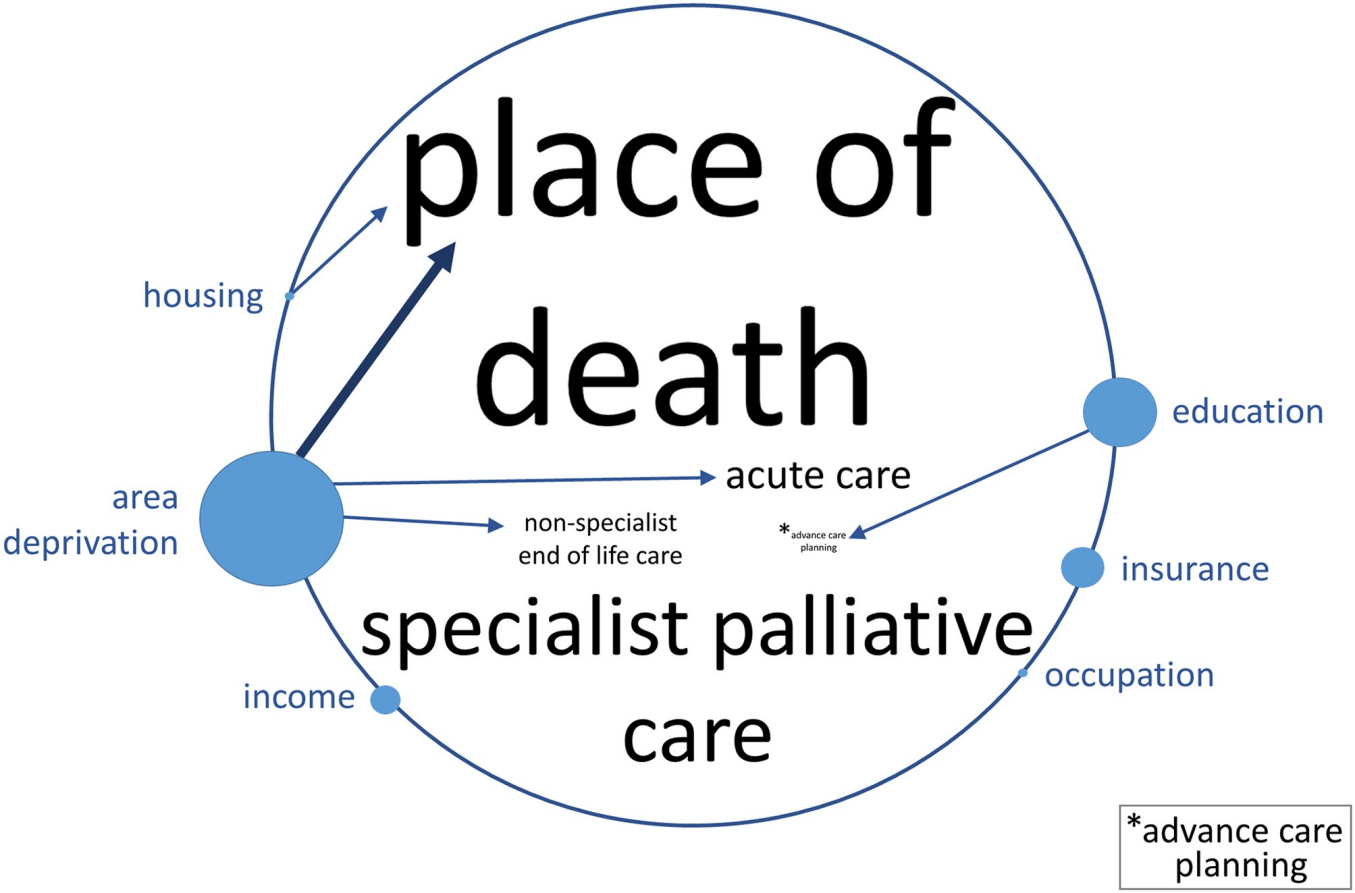
Diagram representing the strength of evidence and direction of association between measures of SEP and use of healthcare in the last year of life. Font size of the outcomes in the centre of the circle, and circle size accompanying the SEP exposures around the circumference, are proportionate to the number of high- and medium-quality studies that the factors were reported in (see S4 Text for underlying numbers and S3 Text for details of studies, outcomes, and exposures). Strength of evidence was determined using the algorithm in Fig 1. A bolder arrow represents strong evidence and a lighter arrow moderate evidence. An arrow from exposure to outcome indicates a pro–high-SEP association such that lowest (compared to highest) SEP was associated with an adverse outcome. There was no evidence of pro–low-SEP associations. Associations with low evidence or with fewer than 4 studies are not depicted. SEP, socioeconomic position.

Fig 3 depicts a summary of evidence from 112 high- and medium-quality studies. We found strong evidence of a pro–high-SEP association between area deprivation and place of death, i.e., people with lowest SEP versus those with highest SEP are more likely to die in hospital compared to home. There was also moderate evidence of a pro–high-SEP association between area deprivation and use of both acute care and nonspecialist end-of-life care. We found moderate evidence of a pro–high-SEP association between education and advance care planning, as well as between housing quality and place of death. Overall, there was no evidence of pro–low-SEP associations.

### Association between area deprivation or education and care received towards the end of life

**Fig 4:**
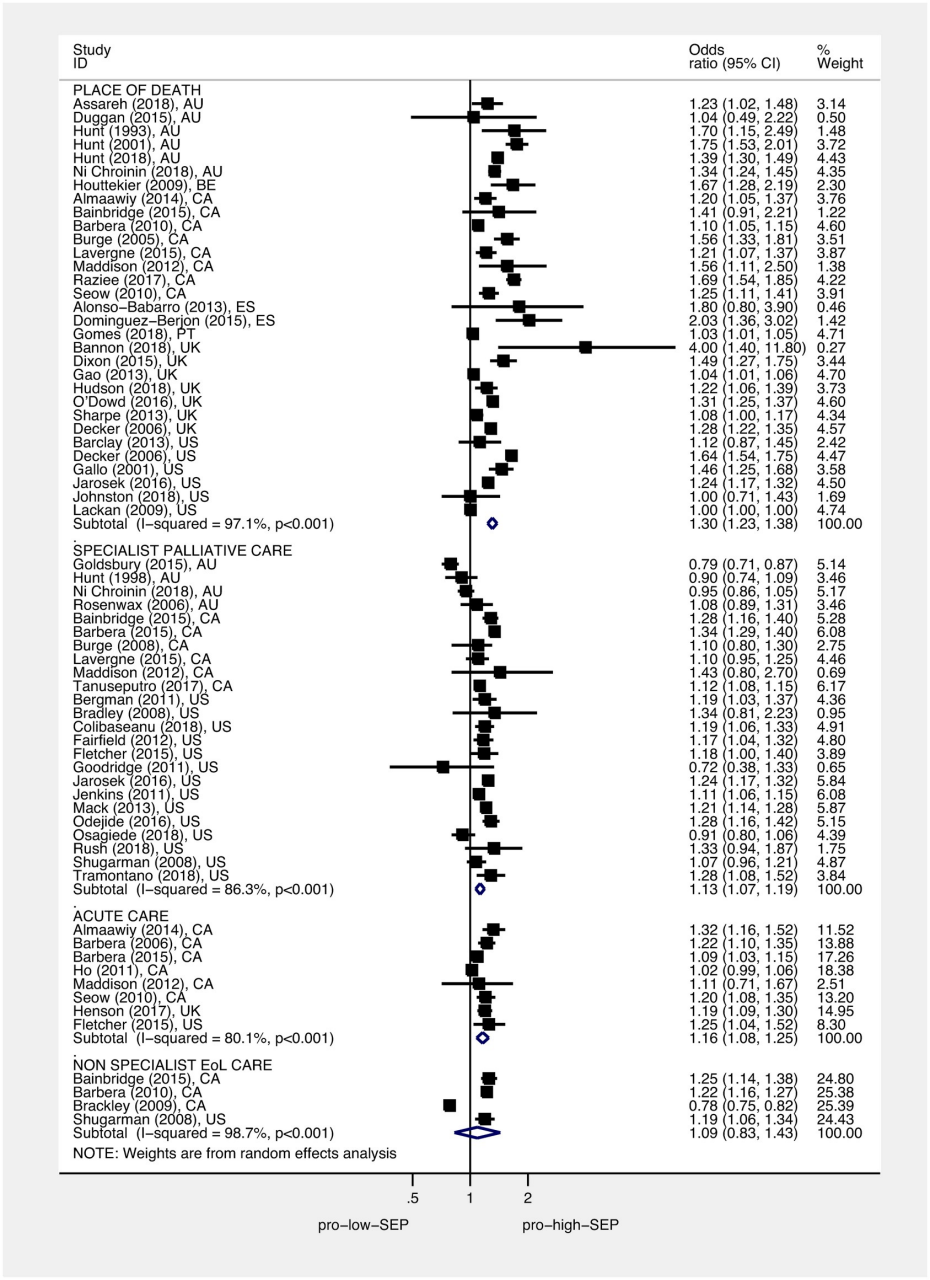
Association between area deprivation and: Place of death, use of acute care, and use of specialist palliative care. Squares show ORs for the most area deprived compared to the least area deprived; diamonds show pooled effects using random-effects models. Place of death (death in hospital versus death at home/hospice/LTC), use of acute care (use of acute services last 3 months of life versus no use), and use of specialist palliative care (not accessing specialist palliative care in the last year of life versus accessing). ORs have been standardised so that >1 indicates that those living in the most deprived areas have higher odds of a worse outcome than those living in the least deprived areas. EoL, end of life; LTC, long-term care; OR, odds ratio; SEP, socioeconomic position.

**Fig 5:**
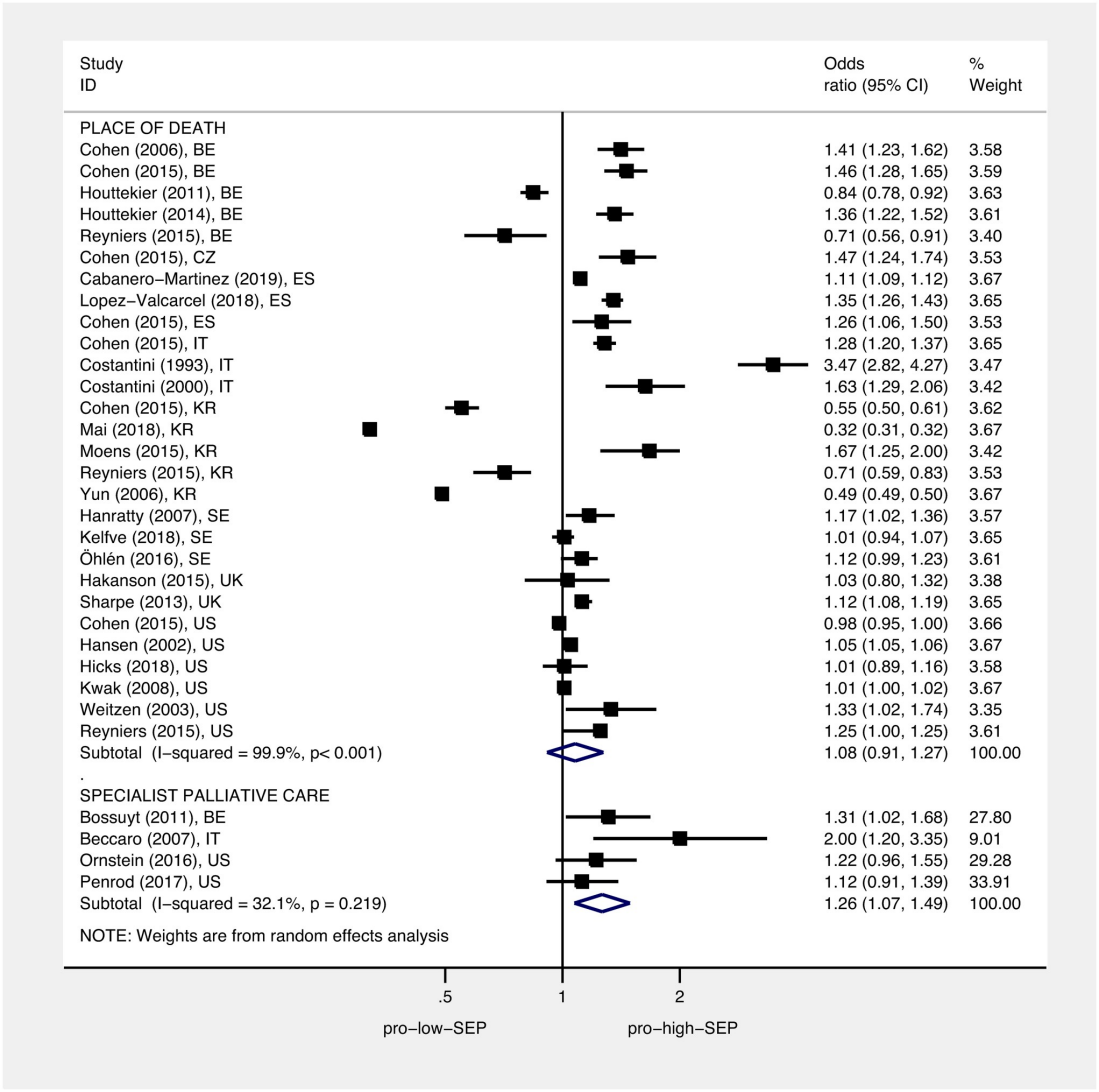
Association between education and: Place of death and use of specialist palliative care. Squares show ORs for the least educated group compared to the most educated group; diamonds show pooled effects using random-effects models. Place of death (death in hospital versus death at home/hospice/LTC) and use of specialist palliative care (not accessing specialist palliative care in the last year of life versus accessing). ORs have been standardised so that >1 indicates that the least educated have higher odds of a worse outcome than the best educated. LTC, long-term care; OR, odds ratio; SEP, socioeconomic position.

**Fig 6:**
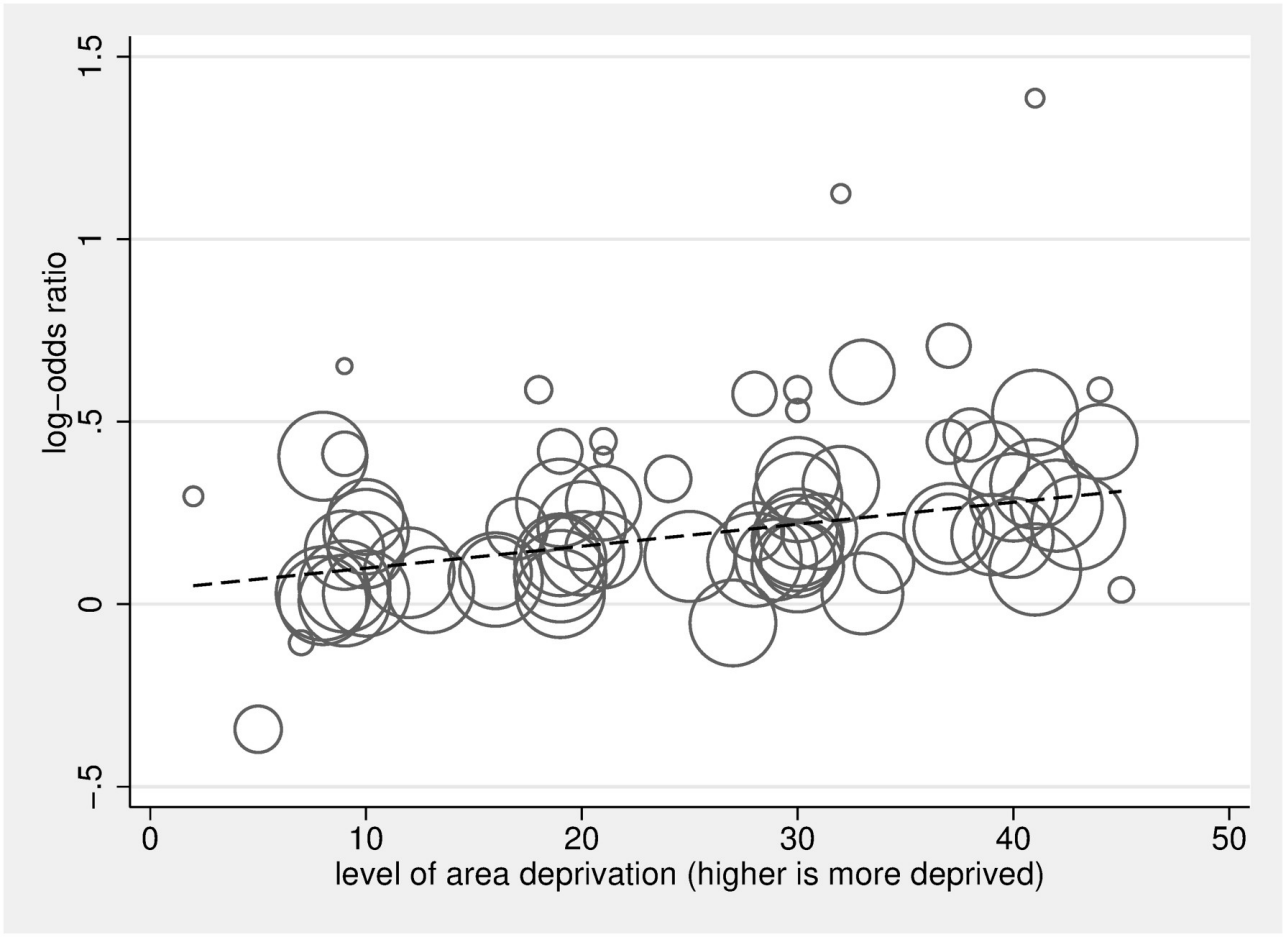
Dose analysis of area deprivation on log-odds of hospital versus home death, compared to the least deprived group. The scatter plot in Fig 6 depicts the linear association between dose of area deprivation (0 being least deprived, 50 being most deprived) and the log-odds of death in hospital versus death at home/hospice/LTC, compared to the least area-deprived group. The circles represent the dose-specific estimates from the 20 included studies [5,21,24,26,34,64,70,86–98]; each study contributes 2, 3, or 4 circles reflecting the number of area-deprivation categories included in the study (the reference category, the least deprived group, is not plotted), and the size of the circle corresponds to the inverse of its total variance. The regression line calculated using the 2-stage glst command in Stata with random effects accounting for within-study dependence reflects a significant positive relationship between dose of area deprivation and likelihood of hospital death (for a 10× unit increase in dose β = 1.07, 95% CI 1.05–1.08, *p* < 0.001). LTC, long-term care.

**Fig 7:**
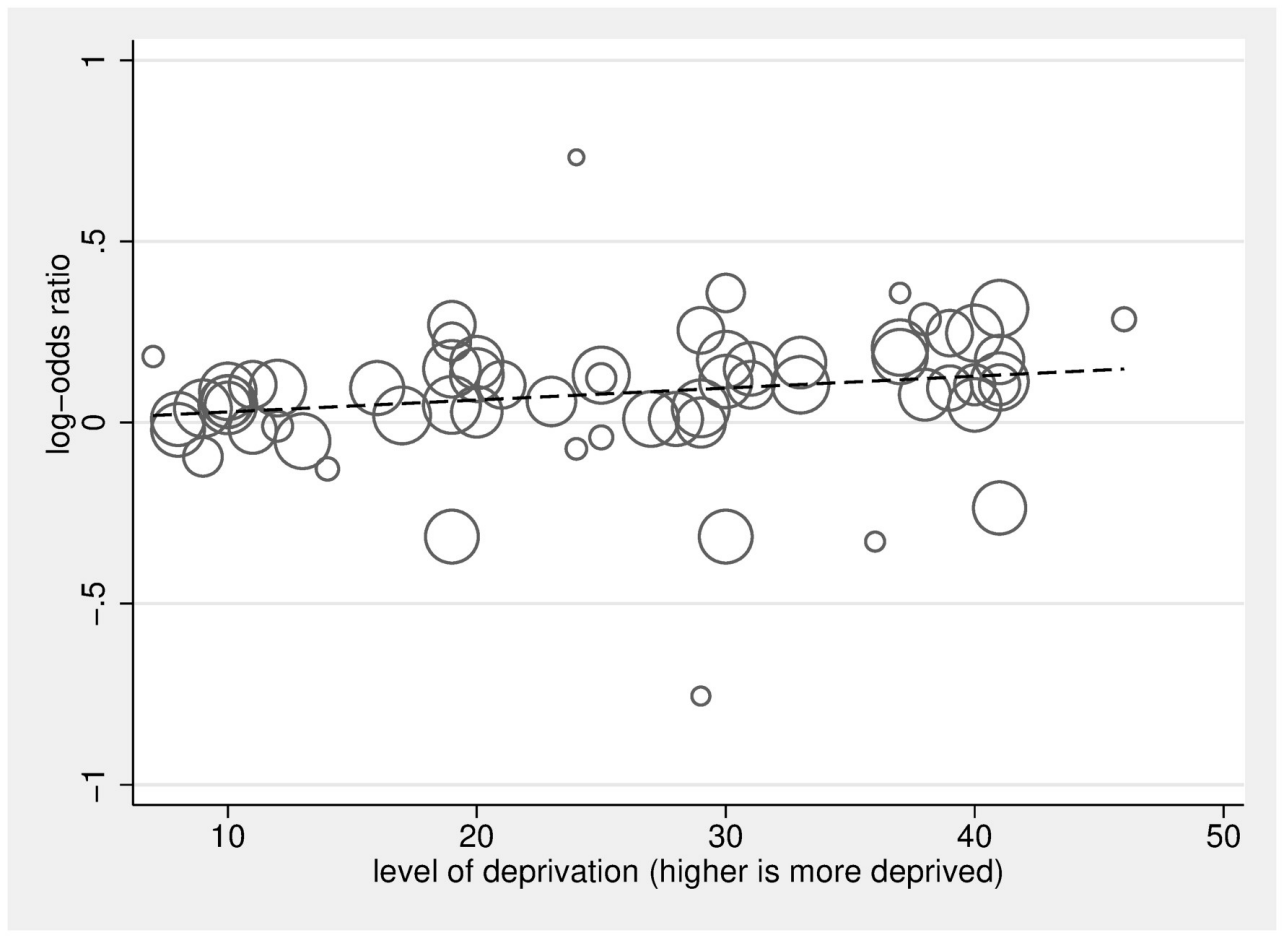
Dose analysis of area deprivation on log-odds of not receiving specialist palliative in the last year of life versus receiving that care, compared to the least deprived group. The scatter plot in Fig 7 depicts the linear association between dose of area deprivation (0 being least deprived, 50 being most deprived) and the log-odds of not receiving specialist palliative care, compared to the least area-deprived group. The circles represent the dose-specific estimates from the 16 included studies [21,32,34,66,67,68,86,98–109]; each study contributes 2, 3, or 4 circles reflecting the number of area-deprivation categories included in the study (the reference category, the least deprived group, is not plotted), and the size of the circle corresponds to the inverse of its total variance. The regression line calculated using the 2-stage glst command in Stata with random effects accounting for within-study dependence reflects a significant positive relationship between dose of area deprivation and likelihood of receiving specialist palliative care (for a 10× unit increase in dose β = 1.03, 95% CI 1.02–1.05, *p* < 0.001).

Figs 4 and 5 display the ORs and 95% CIs for the lowest (most deprived) SEP group compared to the highest (least deprived) SEP group for each study, as well as the pooled ORs using random-effects models. Pooled estimates found that, compared to people living in the least deprived areas, people living in the most deprived areas had an OR of 1.30 for hospital versus home death (95% CI 1.23–1.38, *p* < 0.001), of 1.13 for not receiving specialist palliative care (95% CI 1.07–1.19, *p* < 0.001), of 1.16 for receiving acute hospital-based care in the last 3 months of life (95% CI 1.08–1.25, *p* < 0.001), and of 1.09 for not receiving nonspecialist end-of-life care (95% CI 0.83–1.43, *p* = 0.544). Pooled estimates found that, compared to the most educated, the least educated people had an OR of 1.26 for not receiving specialist palliative care (95% CI 1.07–1.49, *p* = 0.005). Overall, we found no difference between the most educated and the least educated people for odds of hospital versus home death, with an OR of 1.08 (95% CI 0.91–1.27, *p* < 0.377). However, subgroup analysis by country found the pooled estimate for the South Korean studies to be in the opposite direction to other countries (S2 Fig); after omitting the South Korean studies, the pooled estimate for hospital versus home death for the least educated people compared to the most educated people was significant, with an OR of 1.16 (95% CI 1.12–1.21, *p* < 0.001). Heterogeneity was high; *I*^2^ was between 80.1% and 99.9% for all of the subgroup analyses—apart from the subgroup for the association between education and use of specialist palliative care (*I*^2^ = 32.1%, *p* = 0.219). Each of the pooled ORs changed only marginally after omitting successive studies. Dose-response analysis found that, for a 1 quintile (1 unit multiplied by 10, on a 0–50 scale), increase in area deprivation the log-odds of dying in hospital versus home increased by 1.07 (95% CI 1.05–1.08, *p* < 0.001), and log-odds of not receiving specialist palliative care increased by 1.03 (95% CI 1.02–1.05, *p* < 0.001) (Figs 6 and 7, respectively).

## Discussion

This review finds consistent evidence that, in high-income countries, low SEP is associated with adverse healthcare outcomes towards the end of life, including increased odds of hospital versus home death, increased odds of using acute care services in the last 3 months of life, and reduced odds of receiving specialist palliative care in the last year of life. A dose-response association is evident between area deprivation and both place of death and receipt of specialist palliative care, which confirms that inequality persists across the social stratum. Evidence of social inequality in the use of nonspecialist end-of-life care and advance care planning is based on a smaller number of studies and is less conclusive but similarly suggests pro–high-SEP associations. Although awareness of the association between SEP and place of death is longstanding [7,29], this review is the first to synthesise international evidence on social inequality across several components of service use at the end of life.

Most studies used one measure of SEP as a sample descriptor or to control for confounding; area-based measures—and, to a lesser extent, measures of education—dominate. Place of death was the most common outcome studied, reflecting the focus on place of death in end-of-life care research generally and the wide availability of death registry data [110]. Variation in the magnitude of association between different measures of SEP was observed; e.g., area deprivation had a stronger association (OR 1.30) than education (OR 1.08) with place of death, and conversely, education had a stronger association (OR 1.26) than area deprivation (OR 1.13) with use of specialist palliative care. High levels of between-study heterogeneity prevent robust comparisons of the pooled ORs. However, the differences observed raise questions about the relative importance of different aspects of SEP for specific outcomes. For example, it is plausible that level of education acts as a proxy for awareness of specialist palliative care services and that awareness is a stronger driver of access than other aspects of SEP such as area deprivation [14]. The findings from this review support further investigation of these differences.

This review included a comprehensive set of end-of-life outcomes. Eleven studies measuring quality of care using patient-reported or patient-centred outcome measures were found, but all were of low quality and were therefore excluded from meta-analyses. For studies reporting service use before death, assumptions were made about which outcomes are preferable based on existing literature [4,57,62], e.g., that hospital deaths are less favourable than deaths at home or that emergency admission in the last months of life represents an adverse outcome. Subgroup analysis found little variation by country, with the pooled ORs indicating significant pro–high-SEP associations in most countries. The pooled ORs from the South Korean studies on the association between education and place of death, and the Australian studies on the association between area deprivation and access to specialist palliative, are notable exceptions, both suggesting pro–low-SEP inequality albeit with nonsignificant confidence intervals. Different models of care, sociocultural norms around death and dying, and different levels of need are potential explanations for between-country differences. More robust comparison of countries is limited in this review by the small number of studies available from each country.

SEP is a multidimensional construct, as such composite measures combining multiple domains are likely to be the most effective means for capturing statistical variance around SEP [49,50,111]. Area-based indices made up of multiple indicators of SEP are commonly used in this literature and are well suited to monitoring inequality and accounting for confounding from SEP. However, composite measures—area based or otherwise—are less informative about the mechanisms through which SEP might influence outcomes. Area-based measures, whilst being easily linked to patient addresses, are also limited by the ecological fallacy—the assumption that people residing in the same area all share the same deprivation profile. The individual-level measures of SEP commonly used in the studies in this review—education, income, private medical insurance status, or housing tenure—each have benefits and challenges surrounding ease of collection, sufficient sensitivity, and population appropriateness [112]. Essential for a better aetiological understanding of social inequality at the end of life are studies designed specifically for purposes that employ well thought-out measures of SEP and are guided by hypotheses about the relationships between factors [111].

Causal explanations for social patterning in other areas of health have been usefully determined [113]. SEP across the life course influences health through a number of biological, physiological, and environmental mechanisms [114,115, 116]. For care received in the last year of life, proximal social determinants such as ability to pay for care, housing conditions suitable for supporting care at home, understanding and awareness of illness, and availability of services are likely to be important [13]. The cumulative effect of low SEP and worse health—and interrelationships between SEP and other factors known to be important to end-of-life care, such as social support, age, race, and sexuality—are also critical to understand and as yet remain largely unexplored in research. Future studies that use hypothesis-generating qualitative methods and that consider multiple social factors in combination through multilevel and structural models—rather than simply controlling for each as a confounder—could usefully be employed to investigate these relationships.

Evaluation of end-of-life care interventions rarely consider differential outcomes for groups according to SEP, and few interventions have been developed to specifically reduce social inequality in care received towards the end of life; to our knowledge, no review of the effectiveness of current interventions exists. Interventions shown to reduce social inequality outside of end-of-life care offer promising examples, particularly those targeting older people that share similar objectives around managing care in community settings and enhancing quality of life [117,118]. We have shown that social inequality may persist in the care received by people towards the end of life; we must now consider what interventions are effective and begin to target resources at reducing social inequality.

There are some limitations to this review. The first relates to the observational nature of the data included. There was high heterogeneity (*I*^2^) between studies reflecting variation in the measurement of exposures, outcomes, and confounders, as well as in study design and populations. Nevertheless, a major strength is the inclusion of multiple exposure and outcome variables and a large number of observational studies. This necessitated a broad comparison of studies, which ultimately limits the precision of the pooled estimates, even after applying random-effects models. A second limitation is unaccounted confounding from factors related to illness and disease. Many studies included a measure of diagnosis, comorbidity, or disease severity; these varied and were not considered in either the quality evaluation or the analysis. Because people with low SEP experience greater illness and disease—and disease profile also influences patterns of healthcare usage—the inclusion of illness-related factors as a confounder in the association between SEP and the outcomes studied is likely to suppress the social gradient. That being said, illness and disease may also lie on the causal pathway from SEP to the outcomes, making inclusion of illness-related confounders in studies seeking to explore the effect of SEP questionable. Notwithstanding this, the analysis does not consider the important influence of illness and other potential confounders such as service and treatment availability. The third limitation relates to the outcome variables. Assumptions were made about which service-use outcomes are preferable, and these do not take into consideration individual or cultural preferences, need or availability of care, or how these change over time at an individual or societal level. Fourth, this review was limited to studies from high-income countries. The assumptions made around preferable outcomes may not apply to low- and middle-income countries in which healthcare needs and the availability of end-of-life care services are considerably different from those in high-income countries [119]. Important data from low- and middle-income countries is therefore not included. Most of the data were from the US, Canada, and Europe. It is notable that just 7.0% of the included studies were from Asia—of the 682 full-text articles assessed for eligibility, 49 (7.2%) were from Taiwan, 16 (2.3%) from Japan, 14 (2.1%) from South Korea, and 3 (<1%) from Singapore. Therefore, conclusions regarding Asian countries are less strong. A fifth limitation is bias in the identification of studies. The search criteria were necessarily restricted to studies that mentioned SEP in the abstract; on this basis, 2 eligible studies [23,24] from the Henson review [8] used to develop the search strategy were missed from the database search, and it is likely that, on this basis, other studies were missed as well. Considerable effort was made to identify missing studies, hand searching the reference lists of relevant reviews and consulting experts in the field. A further source of bias occurred when eligible studies lacked sufficient information to be included; studies, e.g., [120,121] that did not report an effect size when the association was nonsignificant were not included in the analysis. Finally, given the observational nature of the studies included, we chose to analyse adjusted effects to limit bias from confounding, and we extracted adjusted ORs because these were most commonly used in the literature. However, the outcomes reported are common (>10%), and thus the ORs are overestimations of relative risks; further work to quantify population risk is needed.

## Conclusion

We have found consistent evidence from high-income countries that low SEP is a risk factor across several components of service use at the end of life, including dying in hospital rather than at home, receiving acute hospital-based care in the last 3 months of life, and not receiving specialist palliative care in the last year of life. We also found evidence of a pervasive social gradient in place of death and use of specialist palliative care. These findings should stimulate widespread efforts to reduce socioeconomic inequality towards the end of life. We recommend that all research on care received towards the end of life should attempt to account for SEP, end-of-life care interventions should be analysed for their different effects across the social strata, and the planning and provision of end-of-life care services should consider SEP in local populations.

## Supporting information

S1 PRISMA Checklist PRISMA, Preferred Reporting Items for Systematic Reviews and Meta-Analyses. (DOC)

S1 Text Search terms. (DOCX)

S2 Text Reference list for the 97 low-quality studies, by outcome category. (DOCX)

S3 Text Characteristics of 112 high- and medium-quality studies. (DOCX)

S4 Text Strength of evidence and direction of association between measures of SEP and use of healthcare in the last year of life, from 112 high- and medium-quality studies. SEP, socioeconomic position.
(DOCX)

S1 Fig Histogram of the NOS score for the 209 included studies. NOS, Newcastle-Ottawa Quality Assessment Scale.
(EPS)

S2 Fig Association between education and place of death (death in hospital versus death at home/hospice/LTC) for South Korea and all other countries. Squares show ORs for the least educated group compared to the most educated group; diamonds show pooled effects using random-effects models. LTC, long-term care; OR, odds ratio.
(EPS)

## Abbreviations

LTC: long-term care
NOS: Newcastle-Ottawa Quality Assessment Scale
OR: odds ratio
PRISMA: Preferred Reporting Items for Systematic Reviews and Meta-Analyses
RR: risk ratio
SEP: socioeconomic position
