# Correction: Socioeconomic position and use of healthcare in the last year of life: A systematic review and meta-analysis

**DOI:** 10.1371/journal.pmed.1002878

**Published:** 2019-07-18

**Authors:** Joanna M. Davies, Katherine E. Sleeman, Javiera Leniz, Rebecca Wilson, Irene J. Higginson, Julia Verne, Matthew Maddocks, Fliss E. M. Murtagh

Reference numbers 96 and 118 in the manuscript appear incorrectly, due to reference formatting software errors. They should appear as below:

96. Dixon J, King D, Matosevic T, Clark M, Knapp M. (2015). Equity in Provision of Palliative Care in the UK. Marie Curie: London.

118. Mayhew L, Rickayzen B, Smith D. (2017). Does living in a retirement village extend life expectancy? The case of Whiteley Village. The International Longevity Centre UK: London.
