# Supplementary material for: Socioeconomic position and use of healthcare in the last year of life: A systematic review and meta-analysis
Source: PLoS Med. 2019 Apr 23;16(4):e1002782. doi: 10.1371/journal.pmed.1002782 (PMC6478269; doi:10.1371/journal.pmed.1002782)
Supplement: S1 Text — (DOCX) [file pmed.1002782.s002.docx]

**S2 Text. Search terms**

| **Subject headings** for each database were combined with the key word terms in the table below. Items within columns were combined using OR, the three columns (population, exposure, study type) were combined using AND. | | | |
| --- | --- | --- | --- |
| **Database** | **Population** | **Exposure** | **Study type** |
| **MEDLINE** | Exp Palliative Care/  Exp Terminal Care/  Exp Terminally Ill/  Exp Hospices/ | Exp Socioeconomic Factors/  Exp Residence Characteristics/ | Exp Registries/ |
| **EMBASE** | Exp cancer palliative therapy/  Exp terminal care/  Exp terminally ill patient/  Exp hospice/ | Exp social status/  Exp socioeconomics/  Exp social welfare/ | Exp Registration/ |
| **PsycINFO** | Exp Palliative care/  Exp Hospice/  Exp Terminally Ill Patients/ | Exp socioeconomic status/  Exp disadvantaged/  Exp Economic security/  Exp “INCOME (ECONOMIC)”/  Exp Income Level/  Exp Poverty/ | [no appropriate subject headings] |
| **CINAHL** | (MH “Terminal Care+”)  (MH “Terminally Ill Patients+”)  (MM “Hospice and Palliative Nursing”)  (MM “Hospices”) | (MH “Socioeconomic Factors+”)  (MM “Social Determinants of Health”) | (MH “Registries, Disease”) |
| **ASSIA** | SU.EXACT("Palliative care")  SU.EXACT(“palliative medicine”)  SU.EXACT(“terminal care”)  SU.EXACT(“terminal illness”)  SU.EXACT(“terminally ill people”)  SU.EXACT(“hospices”) | SU.EXACT("Economic conditions")  SU.EXACT("Deprivation")  SU.EXACT("Welfare benefits") |  |

| **Keywords for OVID (MEDLINE, EMBASE, PsychINFO)** | | |
| --- | --- | --- |
| **Population** | **Exposure** | **Study type** |
| palliat*.tw  terminal*.tw  eol*.tw  end of life.tw  end?of?life.tw  hospice*.tw  dying*.tw  supportive care*.tw  life?limit*.tw  life limit*.tw  inoperable*.tw  incurable*.tw  advanc*.tw  progressiv*.tw  agressiv*.tw  end.tw  **adj2**  diagnos*.tw  diseas*.tw  illnes*.tw  cancer*.tw  malignan*.tw  stage*.tw  dementia*.tw  failure*.tw  heart*.tw  last **adj3** life.tw | Soci*.tw  **adj2**  Positi*.tw  clas*.tw  stat*.tw  group*.tw  strat*.tw  differenc*.tw  disparit*.tw  benefit*.tw  occupat*.tw  employment*.tw  level **adj2** education*.tw  literate*.tw  literac*.tw  income*.tw  low?income*.tw  low income*.tw  socio?econ*.tw  socio econ*.tw  socio demograph*.tw  socio?demograph*.tw  depriv*.tw  under?privileg*.tw  under privileg*.tw  pover*.tw  inequalit*.tw  inequit*.tw  welfare*.tw  insurance*.tw  medicaid*.tw  carstairs*.tw  townsend*.tw  jarman.tw  area **adj2** residence.tw  post?code.tw  post code.tw  neighbourhood*.tw | epidemiolog*.tw  cross?section*.tw  cross section*.tw  cohort*.tw  survey*.tw  observational*.tw  retrospectiv*.tw  prospective*.tw  routine?data.tw  routine data.tw  population?based.tw  population based.tw  registr*.tw  **adj2**  cancer.tw  death.tw  disease.tw  national.tw  central.tw  regional.tw  population*.tw  **adj2**  study*.tw  sample*.tw  data*.tw  design*.tw  based.tw  linked **adj3** data.tw  administrative.tw  **adj2**  data.tw  record*.tw |
| **Keywords for CINAHL** | | |
| **Population** | **Exposure** | **Study type** |
| TI palliat* OR AB palliat*  TI terminal* OR AB terminal*  TI eol* OR AB eol*  TI end of life OR AB end of life  TI end#of#life OR AB end#of#life  TI hospice* OR AB hospice*  TI dying* OR AB dying*  TI supportive care* OR AB supportive care*  TI life#limit* OR AB life#limit*  TI life limit* OR AB life limit*  TI inoperable* OR AB inoperable*  TI incurable* OR AB incurable*  TI advanc* OR AB advanc*  TI progressiv* OR AB progressiv*  TI agressiv* OR AB agressiv*  TI end OR AB end  **N2**  TI diagnos* OR AB diagnos*  TI diseas* OR AB diseas*  TI illnes* OR AB illnes*  TI cancer* OR AB cancer*  TI malignan* OR AB malignan*  TI stage* OR AB stage*  TI dementia* OR AB dementia*  TI failure* OR AB failure*  TI heart* OR AB heart*  TI last OR AB last  **N3**  TI life* OR AB life* | TI Soci* OR AB Soci*  **N2**  TI Positi* OR AB Positi*  TI clas* OR AB clas*  TI stat* OR AB stat*  TI group* OR AB group*  TI strat* OR AB strat*  TI differenc* OR AB differenc*  TI disparit* OR AB disparit*  TI benefit* OR AB benefit*  TI occupat* OR AB occupat*  TI employment* OR AB employment*  TI literate* OR AB literate*  TI literac* OR AB iterac*  TI income* OR AB income*  TI low#income* OR AB low#income*  TI low income* OR AB low income*  TI socio#econ* OR AB socio#econ*  TI socio econ* OR AB socio econ*  TI socio demograph* OR AB socio demograph*  TI socio#demograph* OR AB socio#demograph*  TI depriv* OR AB depriv*  TI under#privileg* OR AB under#privileg*  TI under privileg* OR AB under privileg*  TI pover* OR AB pover*  TI inequalit* OR AB inequalit*  TI inequit* OR AB inequit*  TI welfare* OR AB welfare*  TI insurance* OR AB insurance*  TI medicaid* OR AB medicaid*  TI carstairs* OR AB carstairs*  TI townsend* OR AB townsend*  TI jarman OR AB jarman  TI post#code OR AB post#code  TI post code OR AB post code  TI neighbourhood* OR AB neighbourhood*  TI level OR AB level  **N2**  TI Education* OR AB Education*  TI area OR AB area  **N2**  TI residence OR AB residence | TI epidemiolog* OR AB epidemiolog*  TI cross#section* OR AB cross#section*  TI cross section* OR AB cross section*  TI cohort* OR AB cohort*  TI survey* OR AB survey*  TI observational* OR AB observational*  TI retrospectiv* OR AB retrospectiv*  TI prospective* OR AB prospective*  TI routine#data OR AB routine#data  TI routine data OR AB routine data  TI population#based OR AB population#based  TI population based OR AB population based  TI registr* OR AB registr*  **N2**  TI cancer OR AB cancer  TI Death OR AB Death  TI disease OR AB disease  TI national OR AB national  TI central OR AB central  TI regional OR AB regional  TI population* OR AB population*  **N2**  TI study* OR AB study*  TI sample* OR AB sample*  TI data* OR AB data*  TI design* OR AB design*  TI based OR AB based  TI linked OR AB linked  **N3**  TI data OR AB data  TI administrative OR AB administrative  **N2**  TI data OR AB data  TI record* OR AB record* |

| **Search terms for ASSIA**  (((SU.EXACT("Palliative care") OR SU.EXACT(“palliative medicine”) OR SU.EXACT(“terminal care”) OR SU.EXACT(“terminal illness”) OR SU.EXACT(“terminally ill people”) OR SU.EXACT(“hospices”)) OR (ab,ti(palliat*) OR ab,ti(terminal*) OR ab,ti(eol*) OR ab,ti(end of life) OR ab,ti(end?of?life) OR ab,ti(hospice*) OR ab,ti(dying*) OR ab,ti(supportive care*) OR ab,ti(life?limit*) OR ab,ti(life limit*) OR ab,ti(inoperable*) OR ab,ti(incurable*)) OR ((ab,ti(advanc*) OR ab,ti(progressiv*) OR ab,ti(agressiv*) OR ab,ti(end)) WITHIN 2 (ab,ti(diagnos*) OR ab,ti(diseas*) OR ab,ti(illnes*) OR ab,ti(cancer*) OR ab,ti(malignan*) OR ab,ti(stage*) OR ab,ti(dementia*) OR ab,ti(failure*) OR ab,ti(heart*))) OR (ab,ti(last) WITHIN 3 ab,ti(life))) AND ((SU.EXACT("Economic conditions") OR SU.EXACT("Deprivation") OR SU.EXACT("Welfare benefits")) OR (ab,ti(Soci*) WITHIN 2 (ab,ti(positi*) OR ab,ti(clas*) OR ab,ti(stat*) OR ab,ti(group*) OR ab,ti(strat*) OR ab,ti(differenc*) OR ab,ti(disparit*) OR ab,ti(benefit*))) OR (ab,ti(occupat*) OR ab,ti(employment*)) OR (ab,ti(level) WITHIN 2 ab,ti(education)) OR (ab,ti(literate*) OR ab,ti(literac*) OR ab,ti(income*) OR ab,ti(low?income*) OR ab,ti(low income*) OR ab,ti(socio?econ*) OR ab,ti(socio econ*) OR ab,ti(socio demograph*) OR ab,ti(socio?demograph*) OR ab,ti(depriv*) OR ab,ti(under privileg*) OR ab,ti(under?privileg*) OR ab,ti(pover*) OR ab,ti(inequalit*) OR ab,ti(inequit*) OR ab,ti(welfare*) OR ab,ti(insurance*) OR ab,ti(Medicaid*) OR ab,ti(carstairs*) OR ab,ti(townsend*) OR ab,ti(jarman*) OR ab,ti(post?code) OR ab,ti(post code) OR ab,ti(neighbourhood)) OR (ab,ti(area) WITHIN 2 ab,ti(residence))) AND ((ab, ti(epidemiolog*) OR ab,ti(cross?section*) OR ab,ti(cross section*) OR ab,ti(cohort*) OR ab,ti(survey*) OR ab,ti(observational*) OR ab,ti(retrospective*) OR ab,ti(prospective*) OR ab,ti(routine?data) OR ab,ti(routine data) OR ab,ti(population based) OR ab,ti(population?based)) OR (ab,ti(population*) WITHIN 2 (ab,ti(study*) OR ab,ti(sample*) OR ab,ti(data*) OR ab,ti(based) OR ab,ti(design*))) OR (ab,ti(registr*) WITHIN 2 (ab,ti(cancer) OR ab,ti(death) OR ab,ti(disease) OR ab,ti(national) OR ab,ti(central) OR ab,ti(regional))) OR (ab,ti(linked) WITHIN 3 ab,ti(data)) OR (ab,ti(administrative) WITHIN 2 (ab,ti(data) OR ab,ti(record))))) |
| --- |
