# Supplementary material for: Socioeconomic position and use of healthcare in the last year of life: A systematic review and meta-analysis
Source: PLoS Med. 2019 Apr 23;16(4):e1002782. doi: 10.1371/journal.pmed.1002782 (PMC6478269; doi:10.1371/journal.pmed.1002782)
Supplement: S2 Text — (DOCX) [file pmed.1002782.s003.docx]

**S4 Text. Reference list for the 97 low quality studies, by outcome category**

**Place of death: ^1-34^**

**Acute care: ^35-42^**

**Specialist palliative care: ^43-71^**

**Non-specialist palliative care: ^72-74^**

**Advance care planning: ^75-86^**

**Quality of care: ^87-97^**

**References**

1. Alonso-Babarro A, Bruera E, Varela-Cerdeira M, et al. Can this patient be discharged home? Factors associated with at-home death among patients with cancer. Journal of clinical oncology : official journal of the American Society of Clinical Oncology. 2011;29(9):1159-1167.

2. Alturki A, Gagnon B, Petrecca K, et al. Patterns of care at end of life for people with primary intracranial tumors: lessons learned. Journal of Neuro-Oncology. 2014;117(1):103-115.

3. Babazono A, Weiner J, Hamada H, et al. Health policy in transition: terminal care and site of death in Japan. Journal of Health Services & Research Policy. 1998;3(2):77-81.

4. Barbera L, Paszat L, Qiu F. End-of-life care in lung cancer patients in Ontario: Aggressiveness of care in the population and a description of hospital admissions. Journal of Pain and Symptom Management. 2008;35(3):267-274.

5. Bescos Oros M, Parra Moncasi P, Coria Balanzat M, et al. Determinants of the place of death for terminal cancer patients recruited in a home care support team program in Huesca province. [Spanish]. Medicina Paliativa. 2009;16(2):89-94.

6. Burge F, Lawson B, Johnston G. Trends in the place of death of cancer patients, 1992-1997. CMAJ Canadian Medical Association Journal. 2003;168(3):265-270.

7. Cantwell P, Turco S, Brenneis C, et al. Predictors of home death in palliative care cancer patients. J Palliat Care. 2000;16(1):23-28.

8. Cardenas-Turanzas M, Grimes RM, Bruera E, et al. Clinical, sociodemographic, and local system factors associated with a hospital death among cancer patients. Supportive Care in Cancer. 2006;14(1):71-77.

9. Catalan-Fernandez JG, Pons-Sureda O, Recober-Martinez A, et al. Dying of cancer. The place of death and family circumstances. Medical Care. 1991;29(9):841-852.

10. Chambaere K, Rietjens JA, Cohen J, et al. Is educational attainment related to end-of-life decision-making? A large post-mortem survey in Belgium. BMC Public Health. 2013;13:1055.

11. Choo SY, Lee SY, Kim CW, et al. Educational differences in health care utilization in the last year of life among South Korean cancer patients. [Korean]. Journal of preventive medicine and public health = Yebang Uihakhoe chi. 2007;40(1):36-44.

12. Daneault S, Labadie JF. Terminal HIV disease and extreme poverty: a review of 307 home care files. Journal of Palliative Care. 1999;15(1):6-12.

13. Davison D, Johnston G, Reilly P, et al. Where do patients with cancer die in Belfast? Irish Journal of Medical Science. 2001;170(1):18-23.

14. De Conno F, Caraceni A, Groff L, et al. Effect of home care on the place of death of advanced cancer patients. European Journal of Cancer. 1996;32A(7):1142-1147.

15. Escobar Pinzon LC, Claus M, Zepf KI, et al. [Dying in Rhineland-Palatinate (Germany): preferred and actual place of death]. Gesundheitswesen. 2013;75(12):853-858.

16. Hank K, Jurges H. The last year of life in Europe: Regional variations in functional status and sources of support. Ageing & Society. 2010;30(6):1041-1054.

17. Houttekier D, Reyniers T, Deliens L, et al. Dying in hospital with dementia and pneumonia: A nationwide study using death certificate data. Gerontology. 2014;60(1):31-37.

18. Howell DM, Abernathy T, Cockerill R, et al. Predictors of home care expenditures and death at home for cancer patients in an integrated comprehensive palliative home care pilot program. Healthcare Policy = Politiques de sante. 2011;6(3):e73-92.

19. Huang YC, Huang SJ, Ko WJ. Going home to die from surgical intensive care units. Intensive Care Medicine. 2009;35(5):810-815.

20. Jordhoy MS, Saltvedt I, Fayers P, et al. Which cancer patients die in nursing homes? Quality of life, medical and sociodemographic characteristics. Palliative Medicine. 2003;17(5):433-444.

21. Karlsen S, Addington-Hall J. How do cancer patients who die at home differ from those who die elsewhere? Palliative Medicine. 1998;12(4):279-286.

22. Klinkenberg M, Visser G, van Groenou MI, et al. The last 3 months of life: care, transitions and the place of death of older people. Health Soc Care Community. 2005;13(5):420-430.

23. Lee LC, Hu CC, Loh el W, et al. Factors affecting the place of death among hospice home care cancer patients in Taiwan. American Journal of Hospice & Palliative Medicine. 2014;31(3):300-306.

24. Leemans K, Van den Block L, Bilsen J, et al. Dying at home in Belgium: a descriptive GP interview study. BMC Family Practice. 2012;13:4.

25. Maida V. Factors that promote success in home palliative care: a study of a large suburban palliative care practice. J Palliat Care. 2002;18(4):282-286.

26. McCusker J. Where cancer patients die: an epidemiologic study. Public Health Reports. 1983;98(2):170-176.

27. Pritchard RS, Fisher ES, Teno JM, et al. Influence of patient preferences and local health system characteristics on the place of death. SUPPORT Investigators. Study to Understand Prognoses and Preferences for Risks and Outcomes of Treatment. Journal of the American Geriatrics Society. 1998;46(10):1242-1250.

28. Reich O, Signorell A, Busato A. Place of death and health care utilization for people in the last 6 months of life in Switzerland: a retrospective analysis using administrative data. BMC Health Services Research. 2013;13:116.

29. Salomon S, Chuang E, Bhupali D, et al. Race/Ethnicity as a Predictor for Location of Death in Patients With Acute Neurovascular Events. American Journal of Hospice & Palliative Medicine. 2017:1049909116687258.

30. Sims A, Radford J, Doran K, et al. Social class variation in place of cancer death. Palliative Medicine. 1997;11(5):369-373.

31. Taylor JS, Zhang N, Meyer L, et al. When more is less: End-of-life disparities among ovarian cancer patients. Journal of Clinical Oncology Conference. 2016;34(no pagination).

32. Temkin-Greener H, Mukamel DB. Predicting place of death in the program of all-inclusive care for the elderly (PACE): participant versus program characteristics. Journal of the American Geriatrics Society. 2002;50(1):125-135.

33. Fukui S, Fujita J, Tsujimura M, et al. Predictors of home death of home palliative cancer care patients: a cross-sectional nationwide survey. Int J Nurs Stud. 2011;48(11):1393-1400.

34. Ziwary SR, Samad D, Johnson CD, et al. Impact of place of residence on place of death in Wales: an observational study. BMC Palliat Care. 2017;16(1):72.

35. Fulton AT, Gozalo P, Mitchell SL, et al. Intensive care utilization among nursing home residents with advanced cognitive and severe functional impairment. Journal of Palliative Medicine. 2014;17(3):313-317.

36. Gessert CE, Haller IV, Kane RL, et al. Rural-Urban Differences in Medical Care for Nursing Home Residents with Severe Dementia at the End of Life. Journal of the American Geriatrics Society. 2006;54(8):1199-1205.

37. Hanratty B, Jacoby A, Whitehead M. Socioeconomic differences in service use, payment and receipt of illness-related benefits in the last year of life: Findings from the British Household Panel Survey. Palliative Medicine. 2008;22(3):248-255.

38. Henson LA, Gomes B, Koffman J, et al. Factors associated with aggressive end of life cancer care. Supportive Care in Cancer. 2016;24(3):1079-1089.

39. Kwok AC, Semel ME, Lipsitz SR, et al. The intensity and variation of surgical care at the end of life: A retrospective cohort study. The Lancet. 2011;378(9800):1408-1413.

40. Matter-Walstra KW, Achermann R, Rapold R, et al. Days spent in acute care hospitals at the end of life of cancer patients in four Swiss cantons: a retrospective database study (SAKK 89/09). European Journal of Cancer Care. 2016;9:9.

41. Smith AK, McCarthy E, Weber E, et al. Half of older Americans seen in emergency department in last month of life; most admitted to hospital, and many die there. Health affairs (Project Hope). 2012;31(6):1277-1285.

42. Zheng NT, Mukamel DB, Friedman B, et al. The Effect of Hospice on Hospitalizations of Nursing Home Residents. Journal of the American Medical Directors Association. 2015;16(2):155-159.

43. Addington-Hall J, Altmann D. Which terminally ill cancer patients in the United Kingdom receive care from community specialist palliative care nurses? Journal of Advanced Nursing. 2000;32(4):799-806.

44. Bergman J, Kwan L, Fink A, et al. Hospice and emergency room use by disadvantaged men dying of prostate cancer. Journal of Urology. 2009;181(5):2084-2089.

45. Bhatraju P, Friedenberg AS, Uppal A, et al. Factors associated with utilization of an inpatient palliative care consultation service in an urban public hospital. American Journal of Hospice & Palliative Medicine. 2014;31(6):641-644.

46. Chen H, Haley WE, Robinson BE, et al. Decisions for hospice care in patients with advanced cancer. Journal of the American Geriatrics Society. 2003;51(6):789-797.

47. Chen LF, Chang CM, Huang CY. Home-Based Hospice Care Reduces End-of-Life Expenditure in Taiwan: A Population-Based Study. Medicine. 2015;94(38):e1613.

48. Conner NE. Predictive factors of hospice use among Blacks: applying Andersen's Behavioral Model. American Journal of Hospice & Palliative Medicine. 2012;29(5):368-374.

49. Currow DC, Agar M, Sanderson C, et al. Populations who die without specialist palliative care: does lower uptake equate with unmet need? Palliative Medicine. 2008;22(1):43-50.

50. Grande GE, McKerral A, Todd CJ. Which cancer patients are referred to hospital at home for palliative care? Palliative Medicine. 2002;16(2):115-123.

51. Gray JD, Forster DP. Factors associated with utilization of specialist palliative care services: a population based study. Journal of Public Health Medicine. 1997;19(4):464-469.

52. Greiner K, Perera S, Ahluwalia JS. Hospice Usage by Minorities in the Last Year of Life: Results from the National Mortality Followback Survey. Journal of the American Geriatrics Society. 2003;51(7):970-978.

53. Hunt RW, Fazekas BS, Luke CG, et al. The coverage of cancer patients by designated palliative services: a population-based study, South Australia, 1999. Palliative Medicine. 2002;16(5):403-409.

54. Huo J, Du XL, Lairson DR, et al. Utilization of surgery, chemotherapy, radiation therapy, and hospice at the end of life for patients diagnosed with metastatic melanoma. American Journal of Clinical Oncology. 2015;38(3):235-241.

55. Kao YH, Chiang JK. Effect of hospice care on quality indicators of end-of-life care among patients with liver cancer: a national longitudinal population-based study in Taiwan 2000-2011. BMC Palliative Care. 2015;14:39.

56. Lackan NA, Freeman JL, Goodwin JS. Hospice use by older women dying with breast cancer between 1991 and 1996. Journal of Palliative Care. 2003;19(1):49-53.

57. Lackan NA, Ostir GV, Freeman JL, et al. Decreasing variation in the use of hospice among older adults with breast, colorectal, lung, and prostate cancer. Medical Care. 2004;42(2):116-122.

58. Mukamel DB, Caprio T, Ahn R, et al. End-of-life quality-of-care measures for nursing homes: place of death and hospice. Journal of Palliative Medicine. 2012;15(4):438-446.

59. O'Connor TL, Ngamphaiboon N, Groman A, et al. Hospice utilization and end-of-life care in metastatic breast cancer patients at a comprehensive cancer center. Journal of Palliative Medicine. 2015;18(1):50-55.

60. Riggs A, Breuer B, Dhingra L, et al. Hospice Enrollment After Referral to Community-Based, Specialist-Level Palliative Care: Incidence, Timing, and Predictors. Journal of Pain & Symptom Management. 2016;52(2):170-177.

61. Saito AM, Landrum MB, Neville BA, et al. Hospice care and survival among elderly patients with lung cancer. Journal of Palliative Medicine. 2011;14(8):929-939.

62. Schneider M, Zulian GB. Length of stay in palliative care. An observational study. Revue Internationale Francophone de Soins Palliatifs. 2013;28(3):175-180.

63. De Schreye R, Smets T, Annemans L, et al. Applying quality indicators for administrative databases to evaluate end-of-life care for cancer patients in Belgium. Health Aff (Millwood). 2017;36(7):1234-1243.

64. Seaman JB, Bear TM, Documet PI, et al. Hospice and Family Involvement With End-of-Life Care: Results From a Population-Based Survey. American Journal of Hospice & Palliative Medicine. 2016;33(2):130-135.

65. Taylor JS, Brown AJ, Prescott LS, et al. How equal is end-of-life care among gynecologic oncology patients? Gynecologic Oncology. 2015;137:194-195.

66. Teno JM, Kuo S, Gozalo P, et al. Does hospice improve the quality of care for persons dying with dementia? Palliative Medicine. 2010;1):S44.

67. Virnig BA, Kind S, McBean M, et al. Geographic variation in hospice use prior to death. Journal of the American Geriatrics Society. 2000;48(9):1117-1125.

68. Zapka JG, Carter R, Carter CL, et al. Care at the end of life: focus on communication and race. Journal of Aging & Health. 2006;18(6):791-813.

69. Abernethy AP, Currow DC, Fazekas BS. Specialist palliative care needs of whole populations: a feasibility study using a novel approach. Palliative Medicine. 2004;18(3):239-247.

70. Chiang JK, Lee YC, Kao YH. Trend analysis of end-of-life care between hospice and nonhospice groups of cancer patients in Taiwan for 2002-11. Medicine. 2017;96(34):e7825.

71. Zou WY, El-Serag HB, Sada YH, et al. Determinants and Outcomes of Hospice Utilization Among Patients with Advance-Staged Hepatocellular Carcinoma in a Veteran Affairs Population. Dig Dis Sci. 2018;63(5):1173-1181.

72. Cai J, Guerriere DN, Zhao H, et al. Socioeconomic differences in and predictors of home-based palliative care health service use in Ontario, Canada. Int J Environ Res Public Health. 2017;14 (7) (no pagination)(802).

73. Cunningham CM, Hanley GE, Morgan SG. Income inequities in end-of-life health care spending in British Columbia, Canada: A cross-sectional analysis, 2004-2006. International Journal for Equity in Health. 2011;10:12.

74. Grabbe L. The association of functional status and formal home care in the last year of life. GEORGIA STATE UNIVERSITY 1992:126 p-126 p.

75. Chang Y, Huang C, Lin C. Do-not-resuscitate orders for critically ill patients in intensive care. Nursing Ethics. 2010;17(4):445-455.

76. Chang TS, Su YC, Lee CC. Determinants for aggressive end-of-life care for oral cancer patients: a population-based study in an Asian country. Medicine. 2015;94(4):e460.

77. Huang YC, Huang SJ, Ko WJ. Survey of do-not-resuscitate orders in surgical intensive care units. Journal of the Formosan Medical Association. 2010;109(3):201-208.

78. Kao CY, Wang HM, Tang SC, et al. Predictive factors for do-not-resuscitate designation among terminally ill cancer patients receiving care from a palliative care consultation service. Journal of Pain and Symptom Management. 2014;47(2):271-282.

79. McDonald JC, du Manoir JM, Kevork N, et al. Advance directives in patients with advanced cancer receiving active treatment: attitudes, prevalence, and barriers. Support Care Cancer. 2017;25(2):523-531.

80. Oh DY, Kim JH, Kim DW, et al. CPR or DNR? End-of-life decision in Korean cancer patients: A single center's experience. Supportive Care in Cancer. 2006;14(2):103-108.

81. Silveira MJ, Wiitala W, Piette J. Advance directive completion by elderly Americans: A decade of change. Journal of the American Geriatrics Society. 2014;62(4):706-710.

82. Triplett P, Black BS, Phillips H, et al. Content of advance directives for individuals with advanced dementia. Journal of aging and health. 2008;20(5):583-596.

83. Bischoff KE, Sudore R, Miao Y, et al. Advance care planning and the quality of end-of-life care in older adults. J Am Geriatr Soc. 2013;61(2):209-214.

84. Wagner GJ, Riopelle D, Steckart J, et al. Provider communication and patient understanding of life-limiting illness and their relationship to patient communication of treatment preferences. J Pain Symptom Manage. 2010;39(3):527-534.

85. Saeed F, Xing G, Tancredi DJ, et al. Is Annual Income a Predictor of Completion of Advance Directives (ADs) in Patients With Cancer. American Journal of Hospice & Palliative Medicine. 2018:1049909118813973.

86. Yen YF, Huang LY, Hu HY, et al. Association of Advance Directives Completion With the Utilization of Life-Sustaining Treatments During the End-of-Life Care in Older Patients. J Pain Symptom Manage. 2018;55(2):265-271.

87. Baker R, Wu AW, Teno JM, et al. Family satisfaction with end-of-life care in seriously ill hospitalized adults. Journal of the American Geriatrics Society. 2000;48(5 Suppl):S61-69.

88. Curtis J, Patrick DL, Engelberg RA, et al. A measure of the quality of dying and death: Initial validation using after-death interviews with family members. Journal of Pain and Symptom Management. 2002;24(1):17-31.

89. Halpern MT, Urato MP, Kent EE. The health care experience of patients with cancer during the last year of life: Analysis of the SEER-CAHPS data set. Cancer. 2017;123(2):336-344.

90. Jordhoy MS, Fayers P, Loge JH, et al. Quality of life in advanced cancer patients: the impact of sociodemographic and medical characteristics. British Journal of Cancer. 2001;85(10):1478-1485.

91. Kirkendall A, Holland JM, Keene JR, et al. Evaluation of hospice care by family members of Hispanic and non-Hispanic patients. American Journal of Hospice & Palliative Medicine. 2015;32(3):313-321.

92. Lee JJ, Long AC, Curtis JR, et al. The Influence of Race/Ethnicity and Education on Family Ratings of the Quality of Dying in the ICU. Journal of Pain & Symptom Management. 2016;51(1):9-16.

93. Long AC, Engelberg RA, Downey L, et al. Race, income, and education: Associations with patient and family ratings of end-of-life care and communication provided by physicians-in-training. Journal of Palliative Medicine. 2014;17(4):435-447.

94. Melmed GY, Kwan L, Reid K, et al. Quality of life at the end of life: trends in patients with metastatic prostate cancer. Urology. 2002;59(1):103-109.

95. Seow H, Barbera L, Sutradhar R, et al. The trajectory of functional and symptom scores in the last 6 months of life. Supportive Care in Cancer. 2010;18:S172.

96. Smith AK, Cenzer IS, Knight SJ, et al. The epidemiology of pain during the last 2 years of life. Annals of Internal Medicine. 2010;153(9):563-569.

97. Fleming J, Calloway R, Perrels A, et al. Dying comfortably in very old age with or without dementia in different care settings - a representative "older old" population study. BMC Geriatrics. 2017;17(1):222.
