# Supplementary material for: Socioeconomic position and use of healthcare in the last year of life: A systematic review and meta-analysis
Source: PLoS Med. 2019 Apr 23;16(4):e1002782. doi: 10.1371/journal.pmed.1002782 (PMC6478269; doi:10.1371/journal.pmed.1002782)
Supplement: S3 Text — (DOCX) [file pmed.1002782.s004.docx]

**S5 Text. Characteristics of 112 high and medium quality studies**

OR for the lowest (most disadvantaged) versus the highest (least disadvantaged) socioeconomic position (SEP) group have been standardised so that an OR >1 indicates a pro-high-SEP association

**36 studies reporting an association between SEP and use of specialist palliative care (SPC) in the last year of life,** OR >1 indicates lowest (most disadvantaged group) have higher odds of not receiving SPC than highest (least disadvantaged group)

| **study, year of publication and country** | **sample size** | **pt. group** | **time period** | **outcome** | **SEP exposures [number of categories]** | **OR for lowest vs. highest SEP group** |
| --- | --- | --- | --- | --- | --- | --- |
| Bainbridge (2015), CA^1^ | 22262 | cancer | 2006 | last 6 months of life | area-based (inc) [5] | 1.28 (1.16-1.40) |
| Barbera (2015), CA^2^ | 135207 | cancer | 2004/09 | last 6 months of life | area-based (inc) [5] | 1.34 (1.29-1.40) |
| Beccaro (2007), IT^3^ | 1289 | cancer | 2002/03 | last 3 months of life | education (cg) [3] | 2.00 (1.20-3.35) |
| Bergman (2011), US^4^ | 14521 | cancer | 1992/05 | last 6 months of life | area-based (inc) [4] | 1.19 (1.03-1.37) |
| Bossuyt (2011), BE^5^ | 2445 | non-sudden | 2005/07 | last 3 months of life | education (gp) [3] | 1.31 (1.02-1.68) |
| Bradley (2008), US^6^ | 1907 | cancer | 1997/00 | last 12 months of life | area-based (inc) [4] | 1.34 (0.81-2.23) |
| Brody (2008), US^7^ | 722 | all | 2004/06 | after hospitalisation | insurance [3] | 2.86 (0.91-9.09)† |
| Burge (2008), CA^8^ | 7511 | cancer | 1998/03 | any time before death | area-based (inc) [5] | 1.10 (0.80-1.30) |
| Colibaseanu (2018), US^9^ | 86187 | cancer | 2004/13 | last 6 months of life | insurance [6] income [4] area-based (ed) [4] | 1.03 (0.95-1.12) 1.01 (0.90-1.13) 1.19 (1.06-1.33) |
| Fairfield (2012), US^10^ | 8211 | cancer | 2001/07 | any time before death | area-based (inc) [3] insurance [2] | 1.17 (1.04-1.32) 1.39 (1.24-1.56) |
| Fletcher (2015), US^11^ | 6955 | cancer | 2006/12 | any time before death | area-based (ed) [5] | 1.18 (1.00-1.40) |
| Forst (2018), US^12^ | 12437 | cancer | 2002/12 | any time before death | area-based (ed) [n] | 1.15 (1.08-1.23)* |
| Goldsbury (2015), AU^13^ | 45749 | all | 2007 | last 12 months of life | area-based (md) [5] | 0.79 (0.71-0.87) |
| Goodridge (2011), US^14^ | 700 | all | 2008/09 | last 12 months of life | area-based (md) [5] | 0.72 (0.38-1.33) |
| Hunt (1998), AU^15^ | 5673 | cancer | 1990/93 | any time before death | area-based (?) [3] | 0.90 (0.74-1.09) |
| Iwashyna (2002), US^16^ | 331880 | cancer/strokoe | 1993/97 | any time before death | area-based (inc) [n] insurance [2] | 1.01 (1.00-1.01)* 1.44 (1.39-1.52) |
| Jarosek (2016), US^17^ | 46037 | cancer | 2007/08 | any time before death | area-based (inc) [4] insurance [2] | 1.24 (1.17-1.32) 1.52 (1.43-1.59) |
| Jenkins (2011), US^18^ | 178420 | all | 2002/05 | at time of death | area-based (inc) [3] | 1.11 (1.06-1.15) |
| Kwak (2008), US^19^ | 30765 | non-sudden | 2000/02 | last 12 months of life | education [n] | 1.01 (1.00-1.02)* |
| Lavergne (2015), CA^20^ | 23860 | chronic | 2003/09 | at time of death | area-based (md) [5] | 1.10 (0.95-1.25) |
| Mack (2013), US^21^ | 43912 | cancer | 2002/06 | any time before death | area-based (inc) [4] | 1.21 (1.14-1.28) |
| Maddison (2012), CA^22^ | 1201 | cancer | 2001/08 | last 2 months of life | area-based (inc) [3] | 1.43 (0.80-2.70) |
| Neergaard (2013), DK^23^ | 590 | cancer | 2006 | last 3 months of life | income [3] | 0.94 (0.53-1.95) |
| Ni Chroinin (2018), AU^24^ | 34556 | all | 2007 | during hospitalisation | area-based (md) [5] | 0.95 (0.86-1.05) |
| Odejide (2016), US^25^ | 18777 | cancer | 1999/09 | any time before death | area-based (inc) [5] | 1.28 (1.16-1.42) |
| Ornstein (2016), US^26^ | 1567 | all | 2000/11 | any time before death | education [2] | 1.22 (0.96-1.55) |
| Osagiede (2018), US^27^ | 34811 | cancer | 2004/13 | during hospitalisation | income [4] area-based (ed) [4] insurance [6] | 1.08 (0.94-1.24) 0.91 (0.80-1.06) 1.04 (0.94-1.15) |
| Penrod (2017), US^28^ | 3027 | cancer | 2007/11 | during hospitalisation | education [2] insurance [2] | 1.12 (0.91-1.39) 1.36 (1.00-1.87) |
| Rhodes (2013), US^29^ | 5083 | all | 2000/10 | any time before death | insurance [3] | 1.59 (1.28-1.96) |
| Rosenwax (2006), AU^30^ | 12845 | all | 2000/02 | last 12 months of life | area-based (md) [5] | 1.08 (0.89-1.31) |
| Rubens (2018), US^31^ | 81,219 | cancer | 2005/14 | any time before death | insurance [2] income [4] | 0.59 (0.59-0.74) 1.23 (1.23-1.54) |
| Rush (2018), US^32^ | 3166 | IPF | 2006/12 | any time before death | area-based (inc) [4] | 1.33 (0.94-1.87) |
| Shugarman (2008), US^33^ | 13120 | cancer | 1996/99 | last 12 months of life | area-based (inc) [3] insurance [2] | 1.07 (0.96-1.21) 1.06 (0.95-1.19) |
| Sullivan (2018), US^34^ | 21860 | cancer | 2007/13 | any time before death | income [3] | 0.99 (0.93-1.07) |
| Tanuseputro (2017), CA^35^ | 177817 | all | 2010/12 | last 12 months of life | area-based (inc) [5] | 1.12 (1.08-1.15) |
| Tramontano (2018), US^36^ | 3597 | cancer | 2000/13 | any time before death | area-based (md) [5] | 1.28 (1.08-1.52) |
| ** exposure is numerical scale; † Risk Ratio; not enough data to convert to OR* | | | | | | |

**64 studies reporting an association between SEP and place of death,** OR >1 indicates lowest (most disadvantaged) group have higher odds of dying in hospital (versus home/hospice/LTC) than highest (least disadvantaged group)

| **study, year of publication and country** | **sample size** | **pt. group** | **time period** | **SEP exposures [number of categories]** | **OR for lowest vs. highest SEP group** |
| --- | --- | --- | --- | --- | --- |
| Almaawiy (2014), CA^37^ | 9458 | cancer | 2006 | area-based (inc) [5] | 1.20 (1.05-1.37) |
| Alonso-Babarro (2013), ES^38^ | 524 | cancer | 2005 | area-based (md) [5] | 1.80 (0.80-3.90) |
| Assareh (2018), AU^39^ | 25359 | all | 2010/15 | area based (md) [4] | 1.23 (1.02-1.48) |
| Bainbridge (2015), CA^1^ | 1196 | cancer | 2006 | area-based (inc) [5] | 1.41 (0.91-2.21) |
| Bannon (2018), UK^40^ | 362 | cancer | 2011/12 | area based (md) [5] | 4.0 (1.4-11.8) |
| Barbera (2010), CA^41^ | 112398 | cancer | 2000/04 | area-based (inc) [5] | 1.10 (1.05-1.15) |
| Barclay (2013), US^42^ | 61063 | all | 1999/03 | area-based (inc) [5] | 1.12 (0.87-1.45) |
| Blecker (2016), US^43^ | 12205 | all | 1979/11 | occupation [4] | 1.10 (1.08-1.12) |
| Burge (2005), CA^44^ | 13652 | cancer | 1992/97 | area-based (inc) [5] | 1.56 (1.33-1.81) |
| Cabanero-Martinez (2019), ES^45^ | 1336339 | all | 2012/15 | Education [4] | 1.11 (1.09-1.12) |
| Carollo (2018), DK^46^ | 1834437 | all | 1980/14 | Income [3] | 0.74 (0.66-0.84) |
| Cohen (2006), BE^47^ | 55759 | all | 2001 | education [2] | 1.41 (1.23-1.62) |
| Cohen (2015), IT^48^  ES  BE  CZ  US  KR | 73042 13255 16059 24321 554917 68130 | cancer | 2008 | education [4] | 1.28 (1.20-1.37) 1.26 (1.06-1.50) 1.46 (1.28-1.65) 1.47 (1.24-1.74) 0.98 (0.95-1.00) 0.55 (0.50-0.61) |
| Costantini (1993), IT^49^ | 12305 | cancer | 1986/90 | education [4] | 3.47 (2.82-4.27) |
| Costantini (2000), IT^50^ | 17442 | cancer | 1991 | education [4] | 1.63 (1.29-2.06) |
| Decker (2006), UK^51^  US | 59604 51668 | cancer | 1995/98 | area-based (inc) [3] | 1.28 (1.22-1.35) 1.64 (1.54-1.75) |
| Dixon (2015), UK^52^ | 16845 | all | 2013 | area-based (md) [5] | 1.49 (1.27-1.75) |
| Dominguez-Berjon (2015), ES^53^ | 1035 | ALS | 2003/11 | area-based (md) [5] | 2.03 (1.36-3.02) |
| Duggan (2015), AU^54^ | 923 | cancer | 2006/12 | area-based (md) [5] | 1.04 (0.49-2.22) |
| Gallo (2001), US^55^ | 5752 | cancer | 1994 | area-based (inc) [3] | 1.46 (1.25-1.68) |
| Gao (2014), UK^56^ | 13154705 | cancer | 1984/10 | area-based (md) [5] | 1.04 (1.01-1.06) |
| Gomes (2018), PT^57^ | 715727 | all | 2003/12 | area-based (md) [5] | 1.03 (1.01-1.05) |
| Grundy (2004), UK^58^ | 22962 | cancer | 1991 | housing tenure [2] area-based (md) [2] | 1.16 (1.09-1.23) 1.11 (1.04-1.18)α |
| Hakanson (2015), UK^59^ | 82079 | all | 2012 | education [4] | 1.03 (0.80-1.32) |
| Hanratty (2007), SE^60^ | 14517 | all | 2002 | income [5] education [3] | 1.11 (0.92-1.34) 1.17 (1.02-1.36) |
| Hansen (2002), US^61^ | 2317586 | all | 1997 | education [2] | 1.05 (1.05-1.06) |
| Hedinger (2014), CH^62^ | 45990 | all | 2007/08 | education [5] housing tenure [2] | 1.10 (0.94-1.28)‡  1.96 (1.76-2.18) |
| Hicks (2018), US^63^ | 19365 | chronic | 2010/15 | education [8] insurance [6] | 1.01 (0.89-1.16) 1.03 (0.96-1.12) |
| Houttekier (2009), BE^64^ | 3232 | chronic | 2003 | area-based (md) [4] | 1.67 (1.28-2.19) |
| Houttekier (2011), BE^65^ | 79846 | chronic | 1998/07 | education [2] | 0.84 (0.78-0.92) |
| Houttekier (2014), BE^66^ | 42999 | chronic | 2008 | education [3] | 1.36 (1.22-1.52) |
| Huang (2015), TW^67^ | 28978 | cancer | 2009/11 | income [3] | 2.00 (1.92-2.04) |
| Hudson (2018), UK^68^ | 13790 | Liver | 2013/15 | area-based [5] | 1.22 (1.06-1.39) |
| Hunt (1993), AU^69^ | 2715 | cancer | 1990 | area-based (md) [3] | 1.70 (1.15-2.49) |
| Hunt (2001), AU^70^ | 29230 | cancer | 1990/99 | area-based (md) [3] | 1.75 (1.53-2.01) |
| Hunt (2018), AU^71^ | 86257 | cancer | 1990/12 | area-based (md) [5] | 1.39 (1.30-1.49) |
| Jarosek (2016), US^17^ | 46037 | cancer | 2007/08 | area-based (inc) [4] insurance [2] | 1.24 (1.17-1.32) 1.18 (1.10-1.25) |
| Johnson (2018), UK^72^ | 2165 | all | 2009/13 | income (cg) [4] | 0.74 (0.54-1.02) |
| Johnston (2018), US^73^ | 2135 | all | 2000/13 | insurance [3] area-based (inc) [3] | 1.11 (0.83-1.43) 1.00 (0.71-1.43) |
| Kelfve (2018), SE^74^ | 75722 | all | 2013 | education [3] | 1.01 (0.94-1.07) |
| Kessler (2005), UK^75^ | 960 | cancer | 1999/02 | occupation [2] | 2.04 (0.92-4.55) |
| Kuo (2016), TW^76^ | 25816 | cancer | 2005/11 | income (hh) [5] | 1.10 (0.89-1.36) |
| Kwak (2008), US^19^ | 30765 | non-sudden | 2001/02 | education [n] | 1.01 (1.00-1.02)* |
| Lackan (2009), US^77^ | 472382 | all | 1999/01 | area-based (inc) [2] education [n] | 1.00 (1.00-1.00) 1.01 (1.00-1.01)* |
| Lavergne (2015), CA^78^ | 23860 | chronic | 2003/09 | area-based (md) [5] | 1.21 (1.07-1.37) |
| Lee (2017), SG^79^ | 19721 | all | 2004/13 | insurance [3] | 1.02 (0.89-1.14) |
| Lopez-Valcarcel (2018), ES^80^ | 79506 | cancer | 2015 | education [3] | 1.35 (1.26-1.43) |
| Maddison (2012), CA^22^ | 1201 | cancer | 2001/08 | area-based (inc) [3] | 1.56 (1.11-2.50) |
| Mai (2018), KR^81^ | 2358211 | all | 2001/14 | education [4] | 0.32 (0.31-0.32) |
| Moens (2015), KR^82^ | 1565 | parkinson's | 2008 | education [2] | 1.67 (1.25-2.00) |
| Motiwala (2006), CA^83^ | 35155 | all | 2001/02 | area-based (md) [n] | 1.17 (1.10-1.25)* |
| Neergaard (2012), DK^84^ | 569 | cancer | 2006 | income [3] M2 per adult [3] | 1.41 (0.97-2.00)α 1.12 (0.88-1.43) |
| Ni Chroinin (2018), AU^24^ | 34556 | all | 2007 | area-based (md) [5] | 1.34 (1.24-1.45) |
| O'Dowd (2016), UK^85^ | 143627 | cancer | 2004/13 | area-based (md) [5] | 1.31 (1.25-1.37) |
| Öhlén (2016), SE^86^ | 19742 | cancer | 2012 | education [4] | 1.12 (0.99-1.23) |
| Penning (2017), CA^87^ | 11816 | all | 2008/11 | income [3] insurance [2] | 1.22¥ 0.92*** |
| Prioleau (2016), US^88^ | 183 | all | 2012 | insurance [2] | 1.52 (0.68-3.38) |
| Raziee (2017), CA^89^ | 193601 | cancer | 2003/10 | area-based (inc) [5] | 1.69 (1.54-1.85) |
| Reyniers (2015), BE^90^  US  KR | 2816 14844 6033 | dementia | 2008 | education [4] | 0.71 (0.56-0.91) 1.25 (1.00-1.25) 0.71 (0.59-0.83) |
| Seow (2010), CA^91^ | 9018 | all | 2005/06 | area-based (inc) [2] | 1.25 (1.11-1.41) |
| Sharpe (2015), UK^92^ | 106898 | cancer | 2001/09 | area-based (md) [5] car ownership [2] education (ind) [3] NS-SEC [?] home ownership [2] | 1.08 (1.00-1.17) 1.04 (1.03-1.06)† 1.12 (1.08-1.19) 1.02 (1.01-1.03)*† 1.08 (1.05-1.09)† |
| Silveira (2006), US^93^ | 349613 | all | 1989/98 | area-based (inc) [n] | 1.04 (1.02-1.06)* |
| Weitzen (2003), US^94^ | 10122 | chronic | 1993 | education [2] | 1.33 (1.02-1.74) |
| Yun (2006), KR^95^ | 2138463 | all | 1992/01 | education [2] occupation [2] | 0.49 (0.49-0.50) 0.65 (0.64-0.65) |
| ** exposure is numerical; † Risk Ratio; not enough data to convert to OR; ‡ middle SEP group is ref category* | | | | | |
| ¥ *CI not reported p<0.05; *** CI not reported p>0.05; α measure duplicated in another study with the same sample* | | | | | |

**19 studies** **reporting an association between SEP and use of acute care in the last year of life**, OR >1 indicates lowest (most disadvantaged) group have higher odds of using acute care services than highest (least disadvantaged group)

| **study, year of publication and country** | **sample size** | **pt. group** | **time period** | **outcome** | **SEP exposures [number of categories]** | **OR for lowest vs. highest SEP group** |
| --- | --- | --- | --- | --- | --- | --- |
| Almaawiy (2014), CA^37^ | 9467 | cancer | 2006 | hospital admission in last 2 weeks of life | area-based (inc) [5] | 1.32 (1.16-1.52) |
| Bainbridge (2015), CA^1^ | 1196 | cancer | 2006 | ED in last 6 months of life | area-based (inc) [5] | 1.38 (0.91-2.11) |
| Barbera (2006), CA^96^ | 21323 | cancer | 2001 | ED in last 2 weeks of life | area-based (inc) [5] | 1.22 (1.10-1.35) |
| Barbera (2015), CA^2^ | 200285 | cancer | 2004/09 | >1 ED or ICU in last 30 days of life | area-based (inc) [5] | 1.09 (1.03-1.15) |
| Chen (2017), TW^97^ | 2072 | Renal | 2002/12 | ICU in last 30 days of life | income [2] | 0.94 (0.76-1.16) |
| Fletcher (2015), US^11^ | 6955 | cancer | 2006/11 | ICU in last 30 days of life | area-based (ed) [5] | 1.25 (1.04-1.52) |
| Gieniusz (2018), US^98^ | 197 | all | 2015 | ICU during terminal hospital admission | insurance [2] | 1.10 (0.24-5.13)† |
| Goldsbury (2015), AU^13^ | 45749 | all | 2007 | >3 ED in last year of life | area-based (md) [5] | 1.80 (1.63-1.99) |
| Henson (2017), UK^99^ | 124030 | cancer | 2011/12 | >1 ED in last 30 days of life | area-based (md) [5] | 1.19 (1.09-1.30) |
| Ho (2011), CA^100^ | 227161 | cancer | 1993/04 | acute or aggressive care in last 30 days of life | area-based (inc) [5] | 1.02 (0.99-1.06) |
| Hudson (2018), UK^68^ | 11731 | Liver | 2013/15 | hospital admission in last year of life | area-based [5] | 0.98 (0.85-1.12) |
| Kelfve (2018), SE^74^ | 75722 | all | 2013 | >3 hospital admissions in last 3 months | education [3] | 1.01 (0.94-1.07) |
| Maddison (2012), CA^22^ | 1201 | cancer | 2001/08 | >1 ED in last 30 days | area-based (inc) [3] | 1.11 (0.71-1.67) |
| Ni Chroinin (2018), AU^24^ | 34556 | all | 2007 | hospital admission in last year of life | area-based (md) [5] | 1.30 (1.17-1.44) |
| Phongtankuel (2018), US^101^ | 115103 | all | 2012 | hospital admission after hospice disenrollment | income [4] | 1.43 (1.32-1.55) |
| Seow (2010), CA^91^ | 9018 | all | 2005/06 | hospital admission in last 2 weeks of life | area-based (inc) [2] | 1.20 (1.08-1.35) |
| Sharma (2009), US^102^ | 21183 | cancer | 1992/02 | ICU during terminal hospital admission | insurance [2] | 1.15 (1.03-1.27) |
| Sleeman (2018), UK^103^ | 3377 | dementia | 2008/13 | ≥1 ED in last year of life | area-based (md) [5] | 1.27 (1.10-1.45)α |
| Spilsbury (2017), US^104^ | 11875 | all | 2009/10 | rate of ED visits | area-based (md) [5] | 1.25 (1.19-1.32)∞ |
| *† Risk Ratio; not enough data to convert to OR; ∞ Hazard Ratio, could not be converted; α Rate Ratio, could not be converted* | | | | | | |

**11 studies reporting an association between SEP and use of non-specialist end of life care in the last year of life,** OR >1 indicates a pro-rich association where the lowest (most disadvantaged) group have higher odds of not using non-specialist end of life care than highest (least disadvantaged group)

| **study, year of publication and country** | **sample size** | **pt. group** | **time period** | **outcome** | **SEP exposures [number of categories]** | **OR for lowest vs. highest SEP group** |
| --- | --- | --- | --- | --- | --- | --- |
| Bahler (2016), CH^105^ | 11310 | all | 2014 | transitions in the last 6 months of life | insurance [2] | 0.80 (0.69-0.93) |
| Bainbridge (2015), CA^1^ | 22262 | cancer | 2006 | homecare in last 6 months of life | area-based (inc) [5] | 1.25 (1.14-1.38) |
| Barbera (2010), CA^41^ | 112398 | cancer | 2000/04 | homecare in last 6 months of life | area-based (inc) [5] | 1.22 (1.16-1.27) |
| Bossuyt (2011), BE^5^ | 2445 | non-sudden | 2005/07 | >1 transition in the last 3 months of life | education (GP) [3] | 0.66 (0.01-1.08) |
| Brackley (2009), CA^106^ | 98327 | all | 1991/00 | homecare in last 12 months of life | area-based (inc) [5] | 0.78 (0.75-0.82) |
| Brody (2008), US^7^ | 722 | all | 2004/06 | homecare at discharge | insurance [3] | 1.32 (0.65-2.63)† |
| Burge (2005), CA^107^ | 7212 | cancer | 1992/97 | GP home vist in last 180 days of life | area-based (inc) [5] | 1.37 (1.15-1.64) |
| Kelfve (2018), SE^74^ | 51697 | all | 2013 | institutionalisation in last month | education [3] | 1.20 (1.09-1.32) |
| Neergaard (2015), DK^108^ | 599 | cancer | 2006 | <2 GP visits in last 90 days of life | income [3] | 1.18 (1.03-1.35) |
| Sharma (2009), US^102^ | 21183 | cancer | 1992/02 | GP visit during the terminal hospital stay | insurance [2] | 1.20 (1.11-1.32) |
| Shugarman (2008), US^33^ | 13120 | cancer | 1996/99 | homecare in last 12 months of life | insurance [2] area-based (inc) [3] | 1.04 (0.93-1.16) 1.19 (1.06-1.34) |
| *† Risk Ratio; not enough data to convert to OR* | | | | | | |

**4 studies reporting an association between SEP and use of advance care planning (ACP) in the last year of life,** OR >1 indicates lowest (most disadvantaged) SEP group have higher odds of not using ACP than highest (least disadvantaged group)

| **study, year of publication and country** | **sample size** | **pt. group** | **time period** | **outcome** | **SEP exposures [number of categories]** | **OR for lowest vs. highest SEP group** |
| --- | --- | --- | --- | --- | --- | --- |
| Burdsall (2014), US^109^ | 2559 | all | 2008/10 | any formal ACP before death | education [3] income [n] | 1.99¥ 1.04¥* |
| Hanson (1996), US^110^ | 13883 | all | 1986 | living will at time of death | income [n] education [n] | 1.01¥* 1.39¥* |
| Hong (2016), KR^111^ | 53 | all | 2012/13 | AD at admission to hospice | education [2] | 9.59 (1.36-67.79) |
| Muni (2011), US^112^ | 3138 | all | 2003/08 | living will at time of death | area-based (inc) [n] education [n] insurance [2] | 1.08 (0.99-1.19)* 1.12 (1.03-1.23)* 1.89 (1.33-2.63) |
| **exposure is numerical; ¥ CI not reported p<0.05* | | | | | | |

**References**

1. Bainbridge D, Seow H, Sussman J, et al. Factors associated with acute care use among nursing home residents dying of cancer: a population-based study. Int J Palliat Nurs. 2015;21(7):349-356.

2. Barbera L, Seow H, Sutradhar R, et al. Quality of end-of-life cancer care in Canada: a retrospective four-province study using administrative health care data. Current Oncology. 2015;22(5):341-355.

3. Beccaro M, Costantini M, Merlo DF, et al. Inequity in the provision of and access to palliative care for cancer patients. Results from the Italian survey of the dying of cancer (ISDOC). BMC Public Health. 2007;7:66.

4. Bergman J, Saigal CS, Miller DC, et al. Hospice Use and High-Intensity Care in Men

Dying of Prostate Cancer. Arch Intern Med. 2011;171(3):204-210.

5. Bossuyt N, Van den Block L, Cohen J, et al. Is individual educational level related to end-of-life care use? Results from a nationwide retrospective cohort study in Belgium. J Palliat Med. 2011;14(10):1135-1141.

6. Bradley CJ, Clement JP, Lin C. Absence of cancer diagnosis and treatment in elderly Medicaid-insured nursing home residents. J Natl Cancer Inst. 2008;100(1):21-31.

7. Brody AA. The effects of an inpatient palliative care team on mortality, utilization, and cost in a large non-profit teaching hospital. University of California, San Francisco 2008:109 p-109 p.

8. Burge FI, Lawson BJ, Johnston GM, et al. A Population-based Study of Age Inequalities in Access to Palliative Care Among Cancer Patients. Med Care. 2008;46(12):1203-1211.

9. Colibaseanu DT, Osagiede O, Spaulding AC, et al. The Determinants of Palliative Care Use in Patients With Colorectal Cancer: A National Study. American Journal of Hospice & Palliative Medicine. 2018;35(10):1295-1303.

10. Fairfield KM, Murray KM, Wierman HR, et al. Disparities in hospice care among older women dying with ovarian cancer.[Erratum appears in Gynecol Oncol. 2012 Sep;126(3):509-10]. Gynecol Oncol. 2012;125(1):14-18.

11. Fletcher SA, Cronin AM, Zeidan AM, et al. Quality of end-of-life care for the myelodysplastic syndromes: Findings from a large national database. Blood. 2015;126 (23):3287.

12. Forst D, Adams E, Nipp R, et al. Hospice utilization in patients with malignant gliomas. Neuro Oncol. 2018;20(4):538-545.

13. Goldsbury DE, O'Connell DL, Girgis A, et al. Acute hospital-based services used by adults during the last year of life in New South Wales, Australia: a population-based retrospective cohort study. BMC Health Serv Res. 2015;15:537.

14. Goodridge D, Buckley A, Marko J, et al. Home care clients in the last year of life: Is material deprivation associated with service characteristics? J Aging Health. 2011;23(6):954-973.

15. Hunt R, McCaul K. Coverage of cancer patients by hospice services, South Australia, 1990 to 1993. Aust N Z J Public Health. 1998;22(1):45-48.

16. Iwashyna TJ, Chang VW, Zhang JX, et al. The lack of effect of market structure on hospice use. Health Serv Res. 2002;37(6):1531-1551.

17. Jarosek SL, Shippee TP, Virnig BA. Place of Death of Individuals with Terminal Cancer: New Insights from Medicare Hospice Place-of-Service Codes. J Am Geriatr Soc. 2016;64(9):1815-1822.

18. Jenkins TM, Chapman KL, Ritchie CS, et al. Hospice use in Alabama, 2002-2005. J Pain Symptom Manage. 2011;41(2):374-382.

19. Kwak J, Haley WE, Chiriboga DA. Racial differences in hospice use and in-hospital death among Medicare and Medicaid dual-eligible nursing home residents. The Gerontologist. 2008;48(1):32-41.

20. Lavergne M, Lethbridge L, Johnston G, et al. Examining palliative care program use and place of death in rural and urban contexts: a Canadian population-based study using linked data. Rural & Remote Health. 2015;15(2):3134-3146.

21. Mack JW, Chen K, Boscoe FP, et al. Underuse of hospice care by Medicaid-insured patients with stage IV lung cancer in New York and California. J Clin Oncol. 2013;31(20):2569-2579.

22. Maddison AR, Asada Y, Burge F, et al. Inequalities in end-of-life care for colorectal cancer patients in Nova Scotia, Canada. J Palliat Care. 2012;28(2):90-96.

23. Neergaard MA, Jensen AB, Olesen F, et al. Access to outreach specialist palliative care teams among cancer patients in Denmark. J Palliat Med. 2013;16(8):951-957.

24. Ni Chroinin D, Goldsbury DE, Beveridge A, et al. Health-services utilisation amongst older persons during the last year of life: a population-based study. BMC Geriatr. 2018;18(1):317.

25. Odejide OO, Cronin AM, Earle CC, et al. Hospice Use Among Patients With Lymphoma: Impact of Disease Aggressiveness and Curability. J Natl Cancer Inst. 2016;108(1).

26. Ornstein KA, Aldridge MD, Mair CA, et al. Spousal Characteristics and Older Adults' Hospice Use: Understanding Disparities in End-of-Life Care. J Palliat Med. 2016;19(5):509-515.

27. Osagiede O, Spaulding AC, Frank RD, et al. Predictors of palliative treatment in stage IV colorectal cancer. Am J Surg. 2018;14:14.

28. Penrod JD, Garrido MM, McKendrick K, et al. Characteristics of Hospitalized Cancer Patients Referred for Inpatient Palliative Care Consultation. J Palliat Med. 2017;20(12):1321-1326.

29. Rhodes RL, Xuan L, Paulk ME, et al. An examination of end- of-life care in a safety net hospital system: A decade in review. J Health Care Poor Underserved. 2013;24(4):1666-1675.

30. Rosenwax LK, McNamara BA. Who receives specialist palliative care in Western Australia - And who misses out. Palliat Med. 2006;20(4):439-445.

31. Rubens M, Ramamoorthy V, Saxena A, et al. Palliative Care Consultation Trends Among Hospitalized Patients With Advanced Cancer in the United States, 2005 to 2014. American Journal of Hospice & Palliative Medicine. 2018:1049909118809975.

32. Rush B, Berger L, Anthony Celi L. Access to Palliative Care for Patients Undergoing Mechanical Ventilation With Idiopathic Pulmonary Fibrosis in the United States. American Journal of Hospice & Palliative Medicine. 2018;35(3):492-496.

33. Shugarman LR, Bird CE, Schuster CR, et al. Age and gender differences in Medicare expenditures and service utilization at the end of life for lung cancer decedents. Womens Health Issues. 2008;18(3):199-209.

34. Sullivan DR, Ganzini L, Lapidus JA, et al. Improvements in hospice utilization among patients with advanced-stage lung cancer in an integrated health care system. Cancer. 2018;124(2):426-433.

35. Tanuseputro P, Budhwani S, Bai YQ, et al. Palliative care delivery across health sectors: A population-level observational study. Palliat Med. 2017;31(3):247-257.

36. Tramontano AC, Nipp R, Kong CY, et al. Hospice use and end-of-life care among older patients with esophageal cancer. Health Science Reports. 2018;1(9):e76.

37. Almaawiy U, Pond GR, Sussman J, et al. Are family physician visits and continuity of care associated with acute care use at end-of-life? A population-based cohort study of homecare cancer patients. Palliative medicine. 2014;28(2):176-183.

38. Alonso-Babarro A, Astray-Mochales J, Dominguez-Berjon F, et al. The association between in-patient death, utilization of hospital resources and availability of palliative home care for cancer patients. Palliat Med. 2013;27(1):68-75.

39. Assareh H, Stubbs JM, Trinh LTT, et al. Variation in out-of-hospital death among palliative care inpatients across public hospitals in New South Wales, Australia. Intern Med J. 2018;24:24.

40. Bannon F, Cairnduff V, Fitzpatrick D, et al. Insights into the factors associated with achieving the preference of home death in terminal cancer: A national population-based study. Palliative & supportive care. 2018;16(6):749-755.

41. Barbera L, Sussman J, Viola R, et al. Factors Associated with End-of-Life Health Service Use in Patients Dying of Cancer. Healthcare Policy = Politiques de sante. 2010;5(3):e125-143.

42. Barclay JS, Kuchibhatla M, Tulsky JA, et al. Association of hospice patients' income and care level with place of death. JAMA internal medicine. 2013;173(6):450-456.

43. Blecker S, Johnson NJ, Altekruse S, et al. Association of occupation as a physician with likelihood of dying in a hospital. JAMA: Journal of the American Medical Association. 2016;315(3):301-303.

44. Burge F, Lawson B, Johnston G. Where a cancer patient dies: the effect of rural residency. J Rural Health. 2005;21(3):233-238.

45. Cabanero-Martinez MJ, Nolasco A, Melchor I, et al. Place of death and associated factors: a population-based study using death certificate data. Eur J Public Health. 2019;02:02.

46. Carollo A, Verdiell NC, Hale JM, et al. Trends in Hospital Deaths in Denmark from 1980 to 2014, at Ages 50 and Older. J Am Geriatr Soc. 2018;28:28.

47. Cohen J, Bilsen J, Hooft P, et al. Dying at home or in an institution. Using death certificates to explore the factors associated with place of death. Health Policy. 2006;78(2-3):319-329.

48. Cohen J, Pivodic L, Miccinesi G, et al. International study of the place of death of people with cancer: a population-level comparison of 14 countries across 4 continents using death certificate data. Br J Cancer. 2015;113(9):1397-1404.

49. Costantini M, Camoirano E, Madeddu L, et al. Palliative home care and place of death among cancer patients: a population-based study. Palliative medicine. 1993;7(4):323-331.

50. Costantini M, Balzi D, Garronec E, et al. Geographical variations of place of death among Italian communities suggest an inappropriate hospital use in the terminal phase of cancer disease. Public Health. 2000;114(1):15-20.

51. Decker SL, Higginson IJ. A tale of two cities: factors affecting place of cancer death in London and New York. Eur J Public Health. 2007;17(3):285-290.

52. Dixon JK, D. Matosevic, T. Equity in Provision of Palliative Care in the UK. LSE, PSSRU, Marie Curie. 2015.

53. Dominguez-Berjon MF, Esteban-Vasallo MD, Zoni AC, et al. Place of death and associated factors among patients with amyotrophic lateral sclerosis in Madrid (Spain). Amyotrophic Lateral sclerosis & Frontotemporal Degeneration. 2015;17(1-2):62-68.

54. Duggan KJ, Wiltshire J, Strutt R, et al. Patterns of palliative and psychosocial care in stage IV NSCLC in south western Sydney. J Thorac Oncol. 2015;2):S365.

55. Gallo WT, Baker MJ, Bradley EH. Factors associated with home versus institutional death among cancer patients in Connecticut. J Am Geriatr Soc. 2001;49(6):771-777.

56. Gao W, Ho Y, Verne J, et al. Geographical and temporal Understanding In place of Death in England (1984–2010): analysis of trends and associated factors to improve end-of-life Care (GUIDE_Care) – primary research. Health Serv Deliv Res. 2014;2(42).

57. Gomes B, Pinheiro MJ, Lopes S, et al. Risk factors for hospital death in conditions needing palliative care: Nationwide population-based death certificate study. Palliat Med. 2018;32(4):891-901.

58. Grundy E, Mayer D, Young H, et al. Living arrangements and place of death of older people with cancer in England and Wales: a record linkage study. Br J Cancer. 2004;91(5):907-912.

59. Hakanson C, Ohlen J, Morin L, et al. A population-level study of place of death and associated factors in Sweden. Scandinavian journal of public health. 2015;43(7):744-751.

60. Hanratty B, Burstrom B, Walander A, et al. Hospital Deaths in Sweden: Are Individual Socioeconomic Factors Relevant? J Pain Symptom Manage. 2007;33(3):317-323.

61. Hansen SM, Tolle SW, Martin DP. Factors associated with lower rates of in-hospital death. J Palliat Med. 2002;5(5):677-685.

62. Hedinger D, Braun J, Zellweger U, et al. Moving to and dying in a nursing home depends not only on health - an analysis of socio-demographic determinants of place of death in Switzerland. PLoS ONE [Electronic Resource]. 2014;9(11):e113236.

63. Hicks K, Downey L, Engelberg RA, et al. Predictors of Death in the Hospital for Patients with Chronic Serious Illness. J Palliat Med. 2018;21(3):307-314.

64. Houttekier D, Cohen J, Bilsen J, et al. Determinants of the Place of Death in the Brussels Metropolitan Region. J Pain Symptom Manage. 2009;37(6):996-1005.

65. Houttekier D, Cohen J, Surkyn J, et al. Study of recent and future trends in place of death in Belgium using death certificate data: a shift from hospitals to care homes. BMC Public Health. 2011;11:228.

66. Houttekier D, Cohen J, Pepersack T, et al. Dying in hospital: a study of incidence and factors related to hospital death using death certificate data. Eur J Public Health. 2014;24(5):751-756.

67. Huang C-Y, Hung Y-T, Chang C-M, et al. The association between individual income and aggressive end-of-life treatment in older cancer decedents in Taiwan. PLoS One. 2015;10.

68. Hudson B, Round J, Georgeson B, et al. Cirrhosis with ascites in the last year of life: a nationwide analysis of factors shaping costs, health-care use, and place of death in England. The Lancet Gastroenterology & Hepatology. 2018;3(2):95-103.

69. Hunt R, Bonett A, Roder D. Trends in the terminal care of cancer patients: South Australia, 1981-1990. Aust N Z J Med. 1993;23(3):245-251.

70. Hunt RW, Fazekas BS, Luke CG, et al. Where patients with cancer die in South Australia, 1990-1999: a population-based review. Med J Aust. 2001;175(10):526-529.

71. Hunt RW, D'Onise K, Nguyen AT, et al. Where Patients With Cancer Die: A Population-Based Study, 1990 to 2012. J Palliat Care. 2018:825859718814813.

72. Johnson MJ, Allgar V, Chen H, et al. The complex relationship between household income of family caregivers, access to palliative care services and place of death: A national household population survey. Palliat Med. 2018;32(2):357-365.

73. Johnston EE, Muffly L, Alvarez E, et al. End-of-Life Care Intensity in Patients Undergoing Allogeneic Hematopoietic Cell Transplantation: A Population-Level Analysis. J Clin Oncol. 2018:JCO2018780957.

74. Kelfve S, Wastesson J, Fors S, et al. Is the level of education associated with transitions between care settings in older adults near the end of life? A nationwide, retrospective cohort study. Palliat Med. 2018;32(2):366-375.

75. Kessler D, Peters TJ, Lee L, et al. Social class and access to specialist palliative care services. Palliative medicine. 2005;19(2):105-110.

76. Kuo TL, Lin CH, Jiang RS, et al. End-of-life care for head and neck cancer patients: a population-based study. Support Care Cancer. 2016;30:30.

77. Lackan NA, Eschbach K, Stimpson JP, et al. Ethnic differences in in-hospital place of death among older adults in California: Effects of individual and contextual characteristics and medical resource supply. Med Care. 2009;47(2):138-145.

78. Lavergne MR, Lethbridge L, Johnston G, et al. Examining palliative care program use and place of death in rural and urban contexts: a Canadian population-based study using linked data. Rural and remote health. 2015;15(2):3134.

79. Lee YS, Akhileswaran R, Ong EHM, et al. Clinical and Socio-Demographic Predictors of Home Hospice Patients Dying at Home: A Retrospective Analysis of Hospice Care Association's Database in Singapore. J Pain Symptom Manage. 2017;53(6):1035-1041.

80. Lopez-Valcarcel BG, Pinilla J, Barber P. Dying at home for terminal cancer patients: differences by level of education and municipality of residence in Spain. Gac Sanit. 2018;31:31.

81. Mai TTX, Lee E, Cho H, et al. Increasing Trend in Hospital Deaths Consistent among Older Decedents in Korea: A Population-based Study Using Death Registration Database, 2001-2014. BMC Palliat Care. 2018;17(1):16.

82. Moens K, Houttekier D, Van den Block L, et al. Place of death of people living with Parkinson's disease: a population-level study in 11 countries. BMC Palliat Care. 2015;14:28.

83. Motiwala SS, Croxford R, Guerriere DN, et al. Predictors of place of death for seniors in Ontario: A population-based cohort analysis. Canadian Journal on Aging. 2006;25(4):363-371.

84. Neergaard MA, Jensen AB, Sokolowski I, et al. Socioeconomic position and place of death of cancer patients. BMJ Supportive & Palliative Care. 2012;2(2):133-139.

85. O'Dowd EL, McKeever TM, Baldwin DR, et al. Place of Death in Patients with Lung Cancer: A Retrospective Cohort Study from 2004-2013. PLoS ONE [Electronic Resource]. 2016;11(8):e0161399.

86. Ohlen J, Cohen J, Hakanson C. Determinants in the place of death for people with different cancer types: a national population-based study. Acta Oncol. 2016:1-7.

87. Penning M, Cloutier DS, Nuernberger K, et al. "When I Said I Wanted to Die at Home I Didn't Mean a Nursing Home": Care Trajectories at the End of Life. Innovation in Aging. 2017;1(1):igx011.

88. Prioleau PG, Soones TN, Ornstein K, et al. Predictors of Place of Death of Individuals in a Home-Based Primary and Palliative Care Program. J Am Geriatr Soc. 2016;64(11):2317-2321.

89. Raziee H, Saskin R, Barbera L. Determinants of Home Death in Patients With Cancer: A Population-Based Study in Ontario, Canada. J Palliat Care. 2017;32(1):11-18.

90. Reyniers T, Deliens L, Pasman HR, et al. International variation in place of death of older people who died from dementia in 14 European and non-European countries. J Am Med Dir Assoc. 2015;16(2):165-171.

91. Seow H, Barbera L, Howell D, et al. Using more end-of-life homecare services is associated with using fewer acute care services: a population-based cohort study. Med Care. 2010;48(2):118-124.

92. Sharpe KH, Cezard G, Bansal N, et al. Policy for home or hospice as the preferred place of death from cancer: Scottish Health and Ethnicity Linkage Study population cohort shows challenges across all ethnic groups in Scotland. BMJ supportive & palliative care. 2015;5(4):443-451.

93. Silveira MJ, Copeland LA, Feudtner C. Likelihood of home death associated with local rates of home birth: influence of local area healthcare preferences on site of death. Am J Public Health. 2006;96(7):1243-1248.

94. Weitzen S, Teno JM, Fennell M, et al. Factors associated with site of death: a national study of where people die. Med Care. 2003;41(2):323-335.

95. Yun YH, Lim MK, Choi K-S, et al. Predictors associated with the place of death in a country with increasing hospital deaths. Palliat Med. 2006;20(4):455-461.

96. Barbera L, Paszat L, Chartier C. Indicators of poor quality end-of-life cancer care in Ontario. J Palliat Care. 2006;22(1):12-17.

97. Chen B, Fan VY, Yiing-Jenq C, et al. Costs of care at the end of life among elderly patients with chronic kidney disease: patterns and predictors in a nationwide cohort study. BMC Nephrol. 2017;18:1-14.

98. Gieniusz M, Nunes R, Saha V, et al. Earlier Goals of Care Discussions in Hospitalized Terminally Ill Patients and the Quality of End-of-Life Care: A Retrospective Study. American Journal of Hospice & Palliative Medicine. 2018;35(1):21-27.

99. Henson LA, Higginson IJ, Gao W, et al. What factors influence emergency department visits by patients with cancer at the end of life? Analysis of a 124,030 patient cohort. Palliat Med. 2017:269216317713428.

100. Ho TH, Barbera L, Saskin R, et al. Trends in the aggressiveness of end-of-life cancer care in the universal health care system of Ontario, Canada. J Clin Oncol. 2011;29(12):1587-1591.

101. Phongtankuel V, Adelman RD, Trevino K, et al. Association Between Nursing Visits and Hospital-Related Disenrollment in the Home Hospice Population. American Journal of Hospice & Palliative Medicine. 2018;35(2):316-323.

102. Sharma G, Freeman J, Zhang D, et al. Continuity of care and intensive care unit use at the end of life. Arch Intern Med. 2009;169(1):81-86.

103. Sleeman KE, Perera G, Stewart R, et al. Predictors of emergency department attendance by people with dementia in their last year of life: Retrospective cohort study using linked clinical and administrative data. Alzheimer's & dementia : the journal of the Alzheimer's Association. 2018;14(1):20-27.

104. Spilsbury K, Rosenwax L, Arendts G, et al. The Association of Community-Based Palliative Care With Reduced Emergency Department Visits in the Last Year of Life Varies by Patient Factors. Ann Emerg Med. 2017;69(4):416-425.

105. Bahler C, Signorell A, Reich O. Health Care Utilisation and Transitions between Health Care Settings in the Last 6 Months of Life in Switzerland. PLoS ONE [Electronic Resource]. 2016;11(9):e0160932.

106. Brackley M, Penning M. Home-care utilization within the year of death: Trends, predictors and changes in access equity during a period of health policy reform in British Columbia, Canada. Health & Social Care in the Community. 2009;17(3):283-294.

107. Burge FI, Lawson B, Johnston G. Home visits by family physicians during the end-of-life: Does patient income or residence play a role? BMC Palliat Care. 2005;4(1):1.

108. Neergaard MA, Olesen F, Sondergaard J, et al. Are Cancer Patients' Socioeconomic and Cultural Factors Associated with Contact to General Practitioners in the Last Phase of Life? International Journal of Family Medicine Print. 2015;2015:952314.

109. Burdsall TDL. The effects of race, socioeconomic status, and religion on formal end-of-life planning. Dissertation Abstracts International Section A: Humanities and Social Sciences. 2014;74(11-A(E)):No Pagination Specified.

110. Hanson LC, Rodgman E. The use of living wills at the end of life. A national study. Arch Intern Med. 1996;156(9):1018-1022.

111. Hong JH, Kwon JH, Kim IK, et al. Adopting advance directives reinforces patient participation in end-of-life care discussion. Cancer Res Treat. 2016;48(2):753-758.

112. Muni S, Engelberg RA, Treece PD, et al. The influence of race/ethnicity and socioeconomic status on end-of-life care in the ICU. Chest. 2011;139(5):1025-1033.
