# Supplementary material for: Socioeconomic position and use of healthcare in the last year of life: A systematic review and meta-analysis
Source: PLoS Med. 2019 Apr 23;16(4):e1002782. doi: 10.1371/journal.pmed.1002782 (PMC6478269; doi:10.1371/journal.pmed.1002782)
Supplement: S4 Text — SEP, socioeconomic position. (DOCX) [file pmed.1002782.s005.docx]

**S6 Text. Strength of evidence and direction of association between measures of socioeconomic position (SEP) and use of health care in the last year of life, from 112 high and medium quality studies**

|  | **Total number of outcomes reported (n=142)** | | | | | | | | | | | | | | |
| --- | --- | --- | --- | --- | --- | --- | --- | --- | --- | --- | --- | --- | --- | --- | --- |
| **Total number of SEP exposures reported (n=166)** | **PoD**  **n=72 (50.7%)** | | | **Acute care**  **n=19 (13.4%)** | | | **SPC**  **n=36 (25.4%)** | | | **Non-SPC**  **n=11 (7.7%)** | | | **ACP**  **n=4 (2.8%)** | | |
| **Direction of association** | PH | null | PL | PH | null | PL | PH | null | PL | PH | null | PL | PH | null | PL |
| **Income**  **n=17 (10.2%)** | 3 | 3 | 1 | 1 | 1 | 0 | 1 | 4 | 0 | 1 | 0 | 0 | 2 | 0 | 0 |
|  | - | | | - | | | - | | | - | | | - | | |
| **Education**  **n=39 (23.5%)** | 15 | 7 | 5 | 0 | 1 | 0 | 2 | 3 | 0 | 1 | 1 | 0 | 4 | 0 | 0 |
|  | - | | | - | | | - | | | - | | | mod/pro-high | | |
| **Insurance**  **n=23 (13.9%)** | 1 | 5 | 0 | 1 | 1 | 0 | 4 | 6 | 0 | 1 | 2 | 1 | 1 | 0 | 0 |
|  | - | | | - | | |  | | | - | | | - | | |
| **Housing**  **n=4 (2.4%)** | 3 | 1 | 0 | 0 | 0 | 0 | 0 | 0 | 0 | 0 | 0 | 0 | 0 | 0 | 0 |
|  | mod/pro-high | | | - | | | - | | | - | | | - | | |
| **Area deprivation n=79 (47.6%)** | 27 | 6 | 0 | 10 | 4 | 0 | 12 | 13 | 1 | 4 | 0 | 1 | 0 | 1 | 0 |
|  | high/pro-high | | | mod/pro-high | | | - | | | mod/pro-high | | | - | | |
| **Occupation**  **n=4 (2.4%)** | 1 | 2 | 1 | 0 | 0 | 0 | 0 | 0 | 0 | 0 | 0 | 0 | 0 | 0 | 0 |
|  | - | | | - | | | - | | | - | | | - | | |

Strength of evidence: mod (moderate strength evidence, ≥60% agreement between studies and minimum of 3 medium quality studies); high (high strength evidence, ≥70% agreement between studies, and minimum of 3 high quality studies) (see algorithm in figure 1).

Direction of evidence: pro-high (pro high SEP association); pro-low (pro low SEP association)

Associations with <4 studies, or where <60% of studies indicated the same direction of effect are not assigned a strength or direction.

Outcomes on multiple countries presented in the same study are counted separately, otherwise studies reporting outcomes for different subsamples for example for different time periods or subgroups by diagnosis were pooled prior to being counted.
